# Isolation, nucleotide sequencing and genomic comparison of a Novel SXT/R391 ICE mobile genetic element isolated from a municipal wastewater environment

**DOI:** 10.1038/s41598-020-65216-5

**Published:** 2020-05-26

**Authors:** Shannon Slattery, J. Tony Pembroke, John G. Murnane, Michael P. Ryan

**Affiliations:** 1 Department of Chemical Sciences, School of Natural Sciences, Bernal Institute, University of Limerick, Limerick, V94 T9PX Ireland; 2 School of Engineering, University of Limerick, Limerick, V94 T9PX Ireland

**Keywords:** Microbiology, Environmental microbiology, Water microbiology

## Abstract

Integrative Conjugative Elements (ICE’s) of the SXT/R391 family have largely been detected in clinical or environmental isolates of Gammaproteobacteria, particularly *Vibrio* and *Proteus* species. As wastewater treatment plants accumulate a large and diverse number of such species, we examined raw water samples taken from a municipal wastewater treatment plant initially using SXT/R391 family integrase gene-specific PCR probes to detect the presence of such elements in a directed approach. A positive amplification occurred over a full year period and a subsequent Restriction Fragment Length Polymorphism (RFLP) analysis revealed a very limited diversity in the treatment plant examined. Samples demonstrating positive amplification were cultured using *Vibrio* and *Proteus* selective media and PCR amplification tracking was utilized to monitor SXT/R391-ICE family containing strains. This screening procedure resulted in the isolation and identification of a *Proteus mirabilis* strain harbouring an ICE. Whole-genome sequencing of this ICE containing strain using Illumina sequencing technology revealed a novel 81 kb element that contained 75 open reading frames on annotation but contained no antibiotic or metal resistance determinants. Comparative genomics revealed the element contained a conserved ICE core with one of the insertions containing a novel bacteriophage defence mechanism. This directed isolation suggests that ICE elements are present in the environment without apparent selective pressure but may contain adaptive functions allowing survival in particular environments such as municipal wastewater which are reservoirs for large bacterial phage populations.

## Introduction

Wastewater treatment plants (WWTP) accumulate large and diverse numbers of pathogens including viruses, bacteria, fungi, protozoans and helminths. The pathogens present may reflect the common diseases in a community and may be present in numbers as great as 100,000,000 per millilitre^1^, with the most dominant bacteria being Alphaproteobacteria, Thermotogae, Deltaproteobacteria and Gammaproteobacteria^2^. Wastewater influent may also contain many *Proteobacteria* which normally occur in the environment^3^. With the abundance and diversity of microorganisms contained in wastewater, many are carriers of Mobile Genetic Elements (MGEs)^4^.

Integrating Conjugative Elements (ICEs) are self-transmissible MGEs that have the ability to integrate into their host’s chromosome, replicate and transfer by conjugation^5,6^. ICEs present in the environment can rapidly spread in bacterial populations and often carry genes that give the ICE an adaptive advantage to that environmental niche. The SXT/R391 family of ICEs is one of the most studied types of MGE with various sub-types of SXT/R391 elements reported^7^. Type 1 elements have been found in a variety of Gammaproteobacteria species including *Vibrio, Providencia, Proteus, Shewanella, Actionbacillus, Alternomonas, Escherichia coli, Enterovibrio, Photobacterium damselae, Idiomarinaceae bacterium* and *Marinomonas*^8^. They are distinguished by possessing a Type 1 integrase, which allows site-specific integration into the 5′ end of the essential *prfC* gene^9^, integration restores a functioning *prfC* gene and encodes a new hybrid PrfC protein once integrated^9^. Type 2, 3 and 4 ICEs all insert at the 3′ end of the multi-copy tRNA-Ser gene and have been found solely in *Vibrio* species^7,10^. Another type of SXT/R391 ICE integrates into the *pabA* (para-aminobenzoate synthase) gene as with ICE*Sh*95^11^.

The integrase encoding gene of the tyrosine recombinase class is a disguising feature of Type 1 elements. Integrase genes from several different SXT/R391 ICE’s were compared to design specific primers to amplify the gene^12^. Type 1 SXT/R391 ICEs show site-specific integration into the 17 bp integration site at the 5′ end of the *prfC* gene^9,13^ and contain a core of 52 conserved genes that make up the ‘backbone’ of the element with the key functions being integration, excision and regulation^14^. Type 1 elements contain five hotspot regions and five variable regions which allow for gene or gene segments to be inserted, giving the element an array of potential adaptive functions^14^. All ICES characterised to date have been isolated serendipitously or identified from genome sequences There have been reports of SXT/R391-like ICEs from wastewater environments, ICE*Vch*Mex1 (GQ463143.1) isolated from sewage in San Luis Potosi, Mexico^15^ and ICE*She*ChnS12 (AXZL01000060.1), isolated from a wastewater treatment plant in China, 2012^15^. However, these were discovered following analysis rather than using a directed approach. ICE*Vch*Mex1 contains genes in all its hotspots and in one variable region and encodes ampicillin resistance^15^. ICE*She*ChnS12 was found to contain genes in all its hotspot but no genes were contained in the variable regions. The hotspots contained accessory genes such as AAA ATPase and a Restriction Modification System^16^.

In this study, a directed approach was used to detect and isolate ICEs of the SXT/R391 family from a municipal wastewater treatment plant and to characterise one of these isolates as the first ICE detected and isolated from Ireland.

## Materials and Methods

### Bacterial strains, elements and media

The bacterial strains and SXT/R391 elements utilised as part of this study are listed in Table 1. Strains were stored at −80 °C in either Luria-Bertani (LB) broth or M9 minimal media containing 50% (v/v) glycerol.

**Table 1:** List of all bacterial strains, bacterial mobile genetic elements and plasmids used in this study.

Bacterial strain	Genotype/Phenotype	Source
*E. coli* ABll57	F − , *thr-1*, *araC14*, *leuB6*, *_*(*gpt-proA*)62, *lacY1*, *tsx-33*, *qsr’−0*, *glnV44*, *galK2*, *λ-*, *Rac-0*, *hisG4*, *rfbC1*, *mgl-51*, *rpoS396*, *rpsL31*(StrR), *kdgK51*, *xylA5*, *mtl-1*, *argE3*, *thi-1*	*E. coli* genetic stock centre (CGSC), Yale University, New Haven, Connecticut, USA
*E. coli* J53	Rif^r^	
*E. coli* CC118(λpir)	*Δ(ara, leu)7697 araD139 ΔlacX74 galE galK phoA20 thi-1 rpsE rpoB(Rf*^*R*^*) argE(am) recA1 λpir*^+^	Addgene
**Mobile genetic element/Plasmid**	**Genotype**	**Source**
ICER391^40^	Km^r^, Hg^r^	Dr. R. W. Hedges, Royal Postgraduate, Medical School, London
ICER997^32^	Am^r^	Dr. R. W. Hedges, Royal Postgraduate Medical School, London
ICESXT^46^	Sm^r^, Su^r^, Tm^r^	Prof. M.M. Colombo, Universitá La Sapienza, Rome,
ICEpMERPH^52^	Hg^r^, As^r^	National Collection of Industrial Food and Marine Bacteria
pBAM1^53^	Am^r^, Km^r^	Addgene

As^r^: Arsenic resistance, Am^r^: Ampicillin resistance, Rif^r^: Rifampicin resistance Hg^r^: Mercury resistance, Km^r^: Kanamycin resistance, Sm^r^: Streptomycin resistance, Su^r^: Sulfamethoxazole resistance, Tm^r^: Trimethoprim resistance.

### Collection and processing of wastewater sample for detection of ICE containing strains

Composite 24 hr wastewater samples were collected from the inlet works at a municipal wastewater treatment plant, Ireland in May and November 2018.

### Bacterial screening and molecular identification of ICE containing strains

Serial dilutions of the collected and processed samples isolated from wastewater were carried out using a 0.85% Saline solution. Serial dilutions from10^−2^ to 10^−8^ were prepared and samples spread on selective media. CLED media was used for the detection of *Proteus* species and TCBS media for the detection of *Vibrio* species, plates were incubated at 37 °C for 24 hours. Colonies were routinely isolated and inoculated using LB broth, incubated at 37 °C for 24 hours at 200 rpm. The remaining overnight culture was used to prepare glycerol stocks and stored at −80 °C. Replica plating was carried out to identify single colonies containing ICE-like elements via PCR. The streak plate technique was used to isolate a single pure colony from the replica plate.

### PCR

DNA extractions from isolates were performed using the DNeasy Blood & Tissue extraction kit (Qiagen) under the Gram-Negative bacterial protocol for liquid cultures. A PowerSoil DNA extraction kit (Qiagen) was used for solid materials. PCR was carried out using specifically designed primers (Table 2) to amplify the characteristic integrase gene of SXT/R391 family ICE MGEs^12^.

**Table 2:** List of primers used in this study with the gene amplified and the nucleotide sequence^12^.

Primer name	Gene amplified	Nucleotide sequence (5′ to 3′)
IntFor1	Integrase	AAACTAGGGCTGGGCTTATAACATGGCG
IntRev1	Integrase	AAAGATGGCAGCTTGCCGCAACCTC

### Community analysis

PCR-RFLP (Restriction Fragment Length Polymorphism) was carried to determine whether more than one ICE type might be present in the isolated samples. NEB_Cutter^17^ (http://www.labtools.us/nebcutter-v3-0/) was used to select restriction endonucleases that could be used to digest modified integrase genes. The nucleotide sequences of sequenced SXT/R391 ICEs present in the ICEBerg database^8^ and the respective integrase gene sequences were selected to determine what enzymes might be utilised to determine RFLP variation. As a result, three restriction endonucleases were selected, *Hpa*II, *Apo*II and *Tau*I. An *ApoII* site was not present in ICER391 (AY090559.1) *int* amplicons while a *TauI* site was not present in ICER997 (KY433363), ICESXT or ICEpMERPH (MH974755).

PCR, as described above, was carried out on selected isolates containing an amplified integrase gene, ICER391, ICER997 and ICESXT amplicons were used as controls. Additional controls consisted of the use of two different molecular weight markers (NEB 1 kb and NEB 100 kb), uncut PCR product and a control no enzyme “mock” digest. After the PCR, the restriction digest was carried out using 5 µl PCR product, 2 µl Tango Buffer, 0.5 µl of relevant restriction endonuclease and 12.5 µl Milli Q water, bringing the total volume to 20 µl. A cocktail of 15 µl was aliquoted into the PCR tube and 5 µl of the PCR product was then added before the digestion, the tubes were incubated at the appropriate temperatures, *Hpa*II and *Apo*II at 37 °C and *Tau*I at 65 °C. The tubes were incubated for 45 minutes in the thermocycler. To visualize the samples, 3 µl of 5X loading dye was added directly into the digest, 10 µl was loaded onto a 1.5% (w/v) agarose gel in 1X TAE Buffer. The gel was set to run for 1 hour at 100 V and photographed under UV light.

### ICE transfer via triparental mating

To determine if the identified ICE could undergo conjugation tri-parental mating was carried out using the isolated strain as a host strain, pBAM1 as a helper (pBAM1 reference) and *E.coli* J53 as a recipient. The idea was to mark the ICE with a mini Tn5 (Kanamycin resistance) via pBAM1 and then mobilise the marker ICE into *E.coli* J53, a Rif resistant strain. Conjugative mating was carried out following the method by Kristensen *et al*. with some modifications^18^. The donor, helper and recipient strains were grown overnight. Overnight cultures were then centrifuged for 5–10 minutes at 10,000 rpm to collect cells, and washed with fresh LB broth. The cultures were mixed in a proportion of 5:1:1 (donor population was increased) and 50 µl were applied onto 0.45 µm filter on LB Agar plates. The plates were incubated at 37 °C for 24 hours. The cells were then scraped off the surface of the filter and added to a 0.85% saline solution. The culture was diluted with antibiotics for counter selection of the donor and helper cells.

The dilutions were spread onto rifampicin (200 µg.ml^−1^) and kanamycin (50 µg.ml^−1^) supplemented plates and grown at 37 °C for 24 hours. Rifampicin had an MIC of 200 µg.ml^−1^ for the host strain as it was susceptible at this concentration while *E. coli* remained resistant. Controls for the donor, helper and recipient were also prepared. Transconjugants were then tested for the presence of the ICE as described above.

### Genome sequencing & genome annotation

Full genome sequencing was carried out by MicrobesNG (University of Birmingham, UK) using 2 ×250 bp paired-end reads HiSeq Illumina Miseq technology giving 30X genome coverage. The genome sequences were analysed using the MicrobesNG’s automated analysis pipeline, the closest available reference was found using Kraken software (Taxonomic Sequence Classification System)^19^ and reads were mapped using BWA (Burrows-Wheeler Aligner) mem^20^ software to assess the quality of the data. *De novo* assembly of the reads was carried out using SPAdes genome assembler software^21^ and the reads were mapped back to the resulting contigs using BWA mem software. The genome was identified amongst the contigs by using the BLAST tool to investigate the presence of several different ICER391 (AY090559) and ICESXT (AY055428) core scaffold genes (*int*, *jef*, *traLEKBVA*, *setCD*). The sequence data was edited on notepad + + (https://notepad-plus-plus.org/downloads/), submitted to RAST (Rapid Annotation using Subsystem Technology) for annotation (see below), exported as a Genbank and Fasta file and reannotated manually with predicted Orfs blasted via BLASTP to confirm ICE association. Molecular maps were created by inputting the ICE sequence files in GenBank (.gnk) or FASTA (.faa) format into RAST^22^ (http://rast.nmpdr.org/). Gene Graphics was used to create gene representations of hotspot and variable regions by inputting the ICE sequence in GenBank (.gnk) format^23^.

### Phylogenetic analysis

Phylogenetic analysis of fully sequenced SXT/R391 ICEs was performed based on comparison with the concatenated amino acid sequences of 48 to 52 SXT/R391 core ICE gene-encoded proteins on all previously sequenced whole SXT/R391 elements. An unrooted phylogenetic tree was constructed by the maximum-likelihood method based on the Poisson correction model using the MEGAX^24^. Bootstrap analysis with 1000 replications was performed to test the reliability of the tree.

## Results & Discussion

### Targeted isolation of SXT/R391 ICEs

Composite 24 hr raw samples isolated from wastewater (from a municipal wastewater treatment plant) were collected at two different times of the year, May 2018 (summer) and November 2018 (winter).

Samples were plated on genera specific selective media as a significant number of SXT/R391 ICE MGEs have been detected in *Proteus and Vibrio* species^7^. After plates were incubated overnight, colonies of interest were swabbed and inoculated in LB Broth, the overnight cultures were then used for DNA extraction and PCR was performed to detect the presence or absence of an ICE specific integrase gene^12^ indicating the presence of an SXT/R391 ICE MGE containing host. Screening and re-plating (once PCR positive amplicons were detected) were carried out over several weeks.

**Figure 1:**
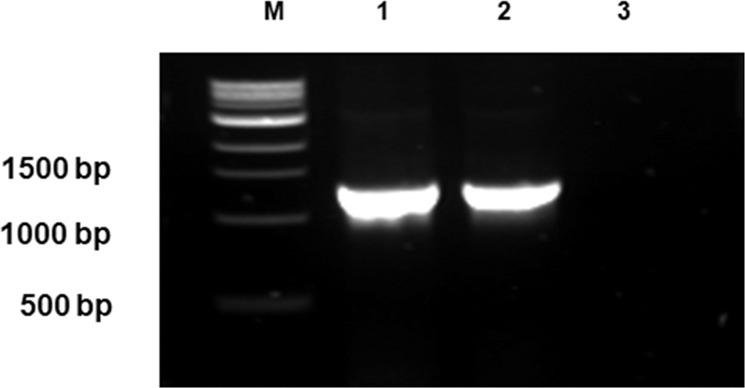
PCR detection of Irish ICE, M: NEB 1 kb Molecular weight marker, 1: ICE*Pmi*Ire01, 2: Positive control (AB1157ICER391) 3: Negative control (dH_2_O as template). SYBR Safe stained on 1.2% agarose gel using primers IntFor1 and IntRev1.

Following multiple screening, and replica plating (see Fig. 1 in Supplementary Material) followed by repeated PCR analysis, the integrase gene was detected by PCR in three translucent blue extracts, ULP001, ULP004 and ULP014 identified on CLED selective media. Translucent blue colonies were screened for the integrase gene and, if detected, samples were streaked out to isolate a single pure colony. A potential SXT/R391-like ICE was detected following further screening from one *Proteus* species, selectively grown on CLED media. A PCR amplicon was detected at 1378 bp matching the positive control, AB1157ICER391, see Fig. 1. From the screened single colony, full sequence analysis of this isolate determined that the host was a *Proteus* species based on analysis of its 16S rDNA sequence. The novel ICE was named *ICEPmi*Ire01 as per standard ICE nomenclature^25^. *Proteus* isolates isolated in the winter also showed positive amplification for the integrase gene, indicating that the presence of SXT/R391 ICE-like elements is apparently not season dependent.

### Community analysis

RFLP was used to determine whether there might be more than one SXT/R391 ICE type present in the wastewater. The integrase specific PCR amplicon generated by IntFor1 and IntRev1 is normally 1378 bp in length. Alignments of numerous ICE integrase genes sequenced from SXT/R391 family members within the ICEberg database^8^ revealed some heterogeneity in gene sequence. Analysis of this heterogeneity using NEBcutter restriction site analysis suggested that using the restriction enzymes *Hpa*II, *Tau*I and *Apo*II could reveal at least some of this RFLP heterogeneity. Therefore, a number of samples were analysed by amplifying putative *int* gene amplicons from wastewater isolates and then subjecting the amplicons to restriction analysis via *Hpa*II, *Tau*I and *Apo*II. We demonstrated that *Hpa*II and *Tau*I were able to cut amplicons generated from samples isolated from wastewater (but not *Apo*II). *Hpa*II cleaved two types of environmental SXT/R391 ICE integrase genes amplified, one without an *Hpa*II site and one with an *Hpa*II site (each amplicon 1378 bp in length), *Tau*I cleaved one SXT/R391 ICE (two amplicons equal to 1378 bp in length) as it contained a *Tau*I site and *Apo*II did not show any cleavage of amplicons from isolates in our case.

**Figure 2:**
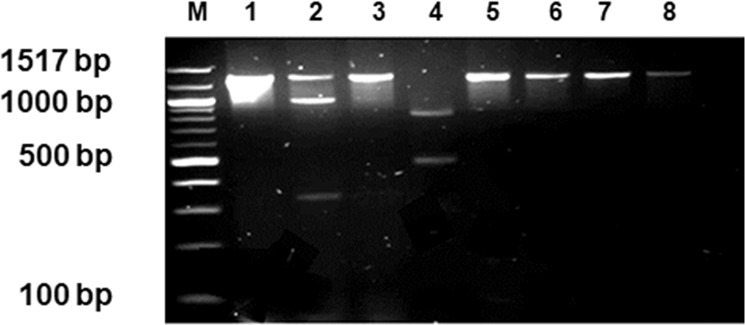
Restriction Fragment length polymorphism of the Proteus isolate, ULP014 (integrase positive), M:100 bp NEB Molecular weight marker, 1: Uncut DNA, 2: ULP014 (*Hpa*II), 3: ULP014 (*Apo*II), 4: ULP014 (*Tau*I), 5: ICER391 (*Hpa*II), 6: ICER391 (*Apo*I), 7: ICER391 (*Tau*I), 8: Control no enzyme “mock digest”.

This limited RFLP yield suggested that only a limited number of amplicon types were present, and indeed possibly only two different types (see Fig. 2, lane 1, where isolate ULP014 when amplified shows two ICE types each at 1378 bp in size, one that contains the *Hpa*II site and one that does not.

### Full sequence analysis

ICE*Pmi*Ire01 (was identified among 271 contigs with a mean fold coverage of 14.98 by searching for the integrase gene and blasting against the prototype ICER391 (AY090559.1) The ICE was found on node 1 (287508 bp in length), this node revealed a novel SXT/R391-like ICE element associated with *Proteus mirabilis*. 16 s rDNA analysis of the host-based on full genome analysis revealed a 100% match to *Proteus mirabilis*. Previously SXT/R391 ICE elements have been detected in *Proteus mirabilis* from China but isolated from clinical and animal samples generally associated with illness^26,27^*. Proteus species* are known to be human and animal opportunistic pathogens, and frequently isolated from urine, wounds and other clinical sites and is present in soil and water habitats possibly from spreading from clinical sources^28^.

**Figure 3:**
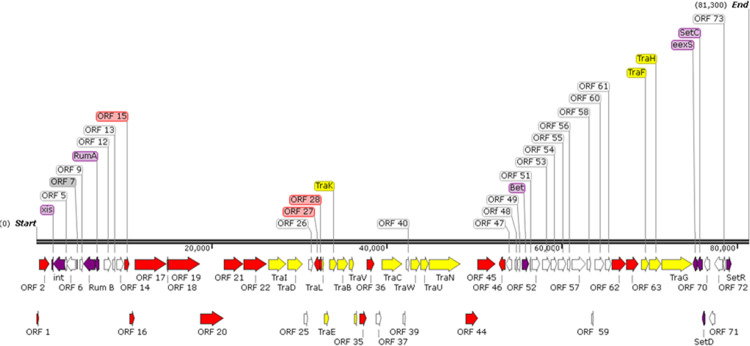
Molecular map of ICE*Pmi*Ire01 showing the location of the genes associated with the 81 kb MGE. Genes coloured purple are associated with excision, integration and control. Genes coloured yellow are associated with conjugative transfer, all other core genes are coloured white. Genes associated with hotspot and variable regions are coloured red (Constructed from MN520463 using Gene Graphics^23^).

This novel SXT/R391 ICE is of the Type 1 ICE variety based on comparative genomics^6^ and is 81 kb in length with prediction of 75 orfs based on RAST annotation. A full genome map can be seen in Fig. 3 Comparative genomics revealed that ICE*Pmi*Ire01 contains 51 core genes, sharing the core molecular backbone and synteny of other SXT/R391 ICEs. A full list of *orfs* detected is presented in Supplementary Table 1 with % similarity to ICER391 and ICESXT illustrated. The core genes of ICE*Pmi*Ire01 are predicted to encode activities for basic ICE functionality such as integration, excision and conjugative transfer^29^. ICE*Pmi*Ire01 is one of the smallest SXT/R391-like ICE MGEs to be discovered thus far, the smallest being ICE*Pmi*Chn3 (KY437727) at 57 kb in length also from a *Proteus mirabilis*, isolated from broiler carcasses in China^26^.

Structural analysis of ICE*Pm*iIre01 revealed that it contains five hotspot regions and further insertions into one variable region. Some of the hotspot regions contain genes putatively encoding resistance to bacteriophage attack. This is not surprising as there is a continuous arms race between bacteria and phage and such resistance systems include restriction-modification systems and toxin-antitoxin systems^30^. Interestingly the element contained no antibiotic resistance or metal resistance determinants.

HS1 contains two predicted genes, *orf27* encoding a putative plasmid-related protein and *orf28* encoding a hypothetical protein with 93% and 97% identity to hypothetical proteins found in ICER391 (AY090559.1). The nucleotide sequence of HS1is 99% similar to that of HS1 of ICER997^31^ but it is unclear what the function of these HS1 predicted genes are.

HS2 contains two genes both putatively associated with the Type IV toxin-antitoxin ‘innate immunity’ bacteria abortive infection (Abi) system that protects bacteria from the spread of phage infection. The Abi system, sometimes denoted as phage exclusion, is characterized by a normal start of bacteriophage infection which is then followed by the interruption of phage development leading to the release of few or no progeny particles but can cause premature host cell death. This Abi system has a survival potential for the population rather than the individual by reducing phage bursts at the expense of individuals within the population^32^. *orf35* encodes a putative AbiEi antitoxin, Type IV TA system with a 96% identity to an Ynd protein (AQT24150.1) found in ICER997 (KY433363.1). *orf36* encodes a putative nucleotidyl transferase AbiEii/AbiGii toxin family protein with a 97% identity to an Ync protein (AQT24151.1) found in ICER997 (KY433363.1). Toxin-antitoxin genes are commonly found in HS2 of SXT/R391-like ICEs as revealed through comparative analysis. The nucleotide sequence of HS2 is 100% identical to that of *Vibrio cholerae*, ICESXT (AY055428).

HS3 contains two genes, none of which show any similarity to ICER391, ICER997 or ICESXT, *orf62* encodes a putative restriction endonuclease, which is 100% identical to a restriction endonuclease (WP_004249392.1) found in Enterobacterial species while *orf63* encodes a putative 5-methylcytosine restriction system component which is 100% identical to the *mcr*BC-5 methylcytosine restriction system of Gammaproteobacteria^33^. The nucleotide sequence of HS3 is 100% identical to that found in ICE*Pmi*Jpn1 (KT894734). These two putative enzymes in HS2 maybe a RMS, which recognizes a specific DNA sequence for cleavage and a cognate methyltransferase that protects from cleavage by methylation of adenine or cytosine bases within the same recognition sequence^34^ and maybe involved in protection from invading phages^35^.

HS4 contains three predicted genes, *orf44* encodes a hypothetical protein with 100% identity to a hypothetical protein (AQT24159.1) found in ICER997 (KY433363.1), *orf45* encodes a putative Helicase HerA-like protein with 100% identity to a Bipolar DNA helicase HerA protein (AQT24160.1) present in ICER997 (KY433363.1). HerA is a DNA helicase able to utilize either 3′ or 5′ single-stranded DNA extensions for loading and subsequent DNA duplex unwinding^36^. Lastly, *orf46* encodes a putative endonuclease protein with a 94% identity to an endonuclease I precursor protein (AQT24161.1) present in ICER997 (KY433363.1). Endonucleases are thought to generate double-strand breaks in DNA. The biological role associated with this hotspot is unknown^37^.

HS5 contains eight predicted genes, six of which make up the novel putative BREX (bacteriophage exclusion) system found in ICE*Pmi*Ire01. This system has characterised homologs that allow for phage adsorption but blocks phage DNA replication. A similar BREX system was first identified in the genome of *Bacillus subtilis* H3081.97 (NZ_ABDL01000007.1) and there confers resistance to a broad range of phage both virulent and temperate^30^. The BREX six gene cassette in *Bacillus subtilis* includes a putative Lon-like protease, a putative alkaline phosphatase protein, a putative RNA-binding protein, a putative DNA methylase, a putative ATPase domain-containing protein and a protein of unknown function^30^. The six genes that make up the BREX system in ICE*Pmi*Ire01 are *orf15*, a putative inner membrane protein (BrxA) that is 100% identical to *orf23*, a hypothetical protein (AAM08040.1) found in ICER391. BrxA shares structural homology with the RNA-binding antitermination protein NusB^31^. The second gene *orf16* is a putative DUF1788 domain-containing protein (BrxB) which is 100% identical to *orf24*, a hypothetical protein (AAM08046.1) contained in ICER391, the function of this gene is unknown^30^. The third gene, *orf17* a putative BREX system P-loop protein (BrxC), is 100% identical to *orf25* a putative ATPase (AAM07998.1) present in ICER391. BrxC contains a putative large ATP binding motif^30^. The fourth gene *orf19* encodes a putative BREX-1 system adenine-specific DNA-methyltransferase (PglX), showing 100% identity to *orf26*, a hypothetical protein (AAM08017.1) found in ICER391. This gene homolog encodes a protein that has putative DNA modifying functions that enable BREX to differentiate between host and phage DNA^30^. The fifth gene is *orf20* encoding a putative BREX-1 system phosphatase protein (PglZ, type A), showing 93% identity to *orf30*, a hypothetical annotated protein (AAM07999.1) present in ICER391. The final gene within the system *orf21* encodes a putative a Lon-related BREX system protease (BrxL), showing 97% identity to *orf31*, a putative ATP-dependent Lon protease protein (AAM08002.1) found in ICER391. Lon proteases are ATP-dependent serine proteases that mediate the selective degradation of mutant and abnormal proteins, as well as certain short-lived regulatory proteins and is required for cellular homeostasis and for survival from DNA damage and development changes induced by stress^38^. Other genes present in the HS5 include *orf18*, encoding a hypothetical protein 100% identical to a hypothetical protein (EKA96589.1) found in *Proteus mirabilis* WGLW6 and *orf22* a putative DNA repair protein showing 100% identity to *orf32*, a hypothetical protein (AAM08000.1) present in ICER391. A similar BREX system appears to be present in ICER391 (AY090559)^39^ and ICE*Vpa*Can1 (CP028481)^7^ based on homology reported above but to our knowledge has not been annotated as a putative BREX system previously. Based on the observations above and based on comparative analysis with other SXT/R391 family members it is clear that ICE*Pmi*Ire01 is a mosaic of core ICE genes and variable genes located in hotspots and variable regions of the element. These genes are most likely accumulated by ICE encoded recombination systems which generated a mosaic-like genetic structure^39^. It is interesting to observe elements of this mosaic structure being found in disparate ICE-like elements isolated from different global locations as outlined above.

ICE*Pmi*Ire01 has an insertion in Variable Region 1 (VR1). Two predicted genes are present *orf01*, encoding a putative transcriptional regulator of the XRE family, which shows 100% identity to a hypothetical protein (AAM08068.1) present in ICER391 (AY090559.1) and *orf02*, encoding a putative Serine/threonine-protein kinase HipA, which is 100% identical to a hypothetical protein (AAM08010.1) also present in ICER391 (AY090559.1). The putative transcriptional regulator of the XRE family, *orf01* is also present in ICE*Bs*1 and is proposed to repress the transcription of genes required for excision and transfer and prevents the acquisition of additional ICE copies, therefore, conferring immunity^40^. HipA-like proteins are expressed by various bacterial species and are involved in high-frequency persistence to the lethal effects of inhibition of either DNA or peptidoglycan. When expressed in high amounts alone, HipA is toxic to bacterial cells^41^ and may also be involved in multidrug tolerance^42^. These genes are commonly associated with VR1 of several other SXT/R391-family ICEs (see comparative genomics section).

ICE*Pmi*Ire01 does not have genes present in variable regions VRII, VRIII, VRIV and VRV as occurs in other SXT\R391 elements and this may be reflect its small size relative to other SXT/R391 ICEs.

### Accession number

The designated GenBank accession number for the nucleotide sequence ICE*Pmi*Ire01 is MN520463.

### Transfer of ICE*Pmi*Ire01

Due to not detecting a selectable marker associated with the ICE it was not possible to demonstrate conjugation of ICE*Pmi*Ire01 in the normal way. Therefore a tri-parnetal mating was undertaken using the ICE*Pmi*Ire01 containing *P. mirabilis* host (susceptible to rifampicin), *E.coli* CC118λ pBAM1, containing a miniTn5 with kanR and *E.coli* J53 as described above. We isolated putative strains of *E. coli* J53 containing ICE*Pmi*Ire01 containing the transposed miniTn5. Transconjugants were then analysed to validate and confirm the conjugative transfer. Transfer frequency of the element can be seen in Table 3.

**Table 3:** Transfer of frequency- calculated per donor cell.

Donor	Helper	Recipient	Frequency of transfer
*Proteus mirabilis* (ICE*Pmi*Ire01) 10^7^	*E.coli* CC118λpir (pBAM1)	*E. coli* (J53)	2.8 × 10^−5^

### Phylogenetic analysis

**Figure 4:**
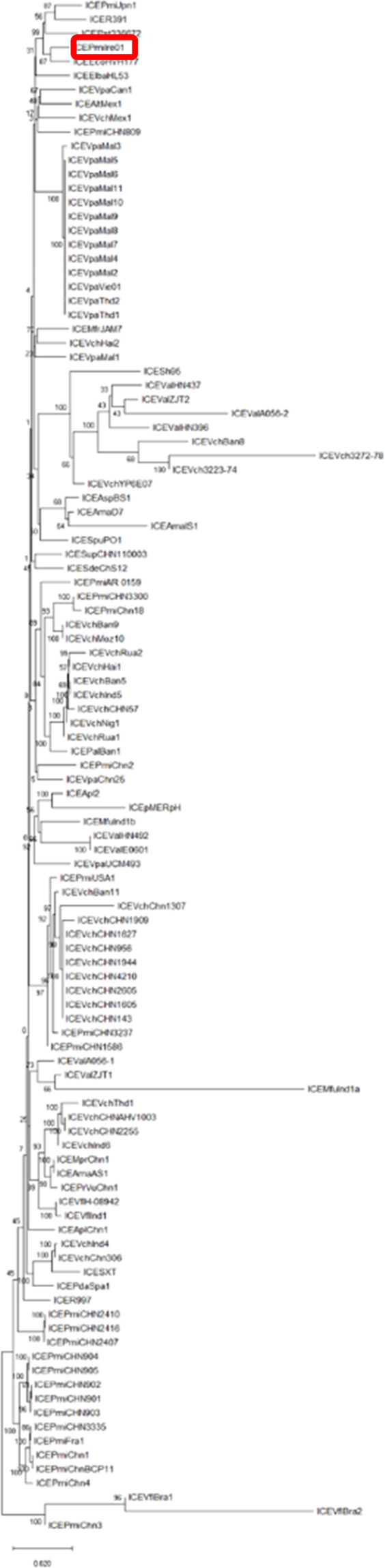
Phylogenetic tree from the maximum-likelihood analysis of the core concatenated proteins of 112 SXT/R391 ICEs including the novel isolate ICE*Pmi*Ire01 illustrating its relationship with other family members.

A phylogenetic tree (Fig. 4) was constructed based on the concatenated amino acid sequences of all SXT-R391 core proteins for all published core genome sequences of these elements. ICE*Pmi*Ire01 is clustered with ICE*Pmi*Jpn1, ICER391, ICE*IdB*USA1 and ICE*PrSt*33672. These results illustrate the wide geographic spread of Type 1 SXT/R391 ICE-like elements with these near neighbours being isolated in Japan, South Africa and the USA respectively. Information on SXT/R391 element in Fig. 4 can be found in Supplementary Table 4.

### Comparative analysis: bacteriophage defence mechanisms

As a result of the abundance of bacteriophage present in wastewater and other environmental niches, bacteria have developed various mechanisms to resist their attack. These resistance mechanisms include RMS, toxin-antitoxin (T-A) system, Abi systems^35^ and BREX systems^30^. The putative BREX system located in HS5 of ICE*Pmi*Ire01 is related to a novel bacteriophage defence system first reported by Goldfarb *et al*. (2015)^30^. It consists of a six gene cassette which includes a putative Lon-like protease, an alkaline phosphatase domain protein, a putative RNA-binding protein, a DNA methylase, an ATPase-domain protein and a protein of unknown function. This system shows high levels of homology and synteny with the putative ICE*Pmi*Ire01 system.

**Figure 5:**
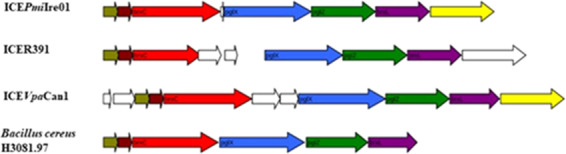
Comparative analysis of the BREX systems present in ICE*Pmi*Ire01, ICER391 and ICE*Vpa*Can1, located in HS5 of these ICE elements. Genes coloured moss are associated with brxA (predicted RNA-binding domain), genes coloured brown associated with brxB (unknown function), genes coloured light blue are associated with pglX (DNA methylation domain), genes coloured dark green are associated with pglZ (alkaline phosphatase domain) and brxL (lon-like protease domain), genes coloured yellow are associated with a putative DNA repair and genes coloured white of unknown function.

By comparing each gene in the putative ICE*Pmi*Ire01 BREX system it was discovered that a near-identical unannotated system was also located in HS5 of ICER391 the prototype SXT/R391 ICE (showing between 93–100% homology)^39^. A recently discovered SXT/R391 ICE, ICE*Vpa*Can1, was also found to contain this putative system^7^ with homology levels between 68–99%. A comparison between the putative HS5 BREX system of ICE*Pmi*Ire01 demonstrated high (84–100%) homology to the BREX system found in *Bacillus cereus* H3081.9. This is most surprising as SXT/R391 ICEs are exclusively found in Gram-negative bacteria and finding a homologous system in a Gram-positive organism with such high levels of homology is unusual (Fig. 5).

**Figure 6:**
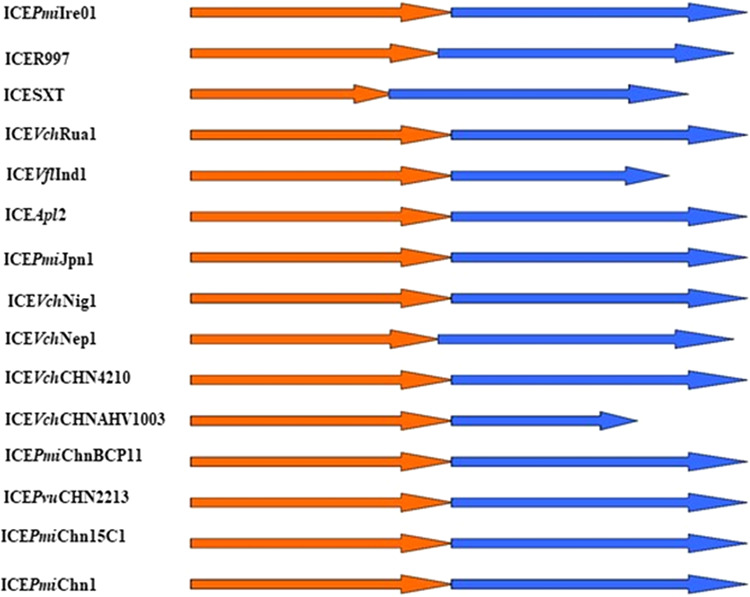
Examples of the genetic organization of HS2 of SXT/R391 ICE elements containing a T-A system, including ICE*Pmi*Ire01, ICER997^31^, ICESXT^45^, ICE*Vch*Rua1^46^, ICE*Vfl*Ind1^14^, ICE*Apl*2^47^, ICE*Pmi*Jpn1^48^, ICE*Vch*Nig1^49^, ICE*Vch*Nep1^49^, ICE*Vch*CHN4210^50^, ICE*Vch*CHNAHV1003^50^, ICE*Pmi*ChnBCP11^51^, ICE*Pvu*CHN2213^27^, ICE*Pmi*Chn15C1 (direct submission) and ICE*Pmi*Chn1^48^. Genes coloured orange are associated with *ynd* and genes coloured blue are associated with *ync*. Both genes encode a toxin-antitoxin system.

The insertion in HS2 was also compared to ICER391 as it contains a putative T-A system and is commonly associated with this hotspot in other ICEs. T-A systems consist of a stable toxin and its labile antitoxin^43^ that promote the maintenance of SXT/R391 ICEs, genes associated with this function are *mos*T and *mos*A (*ynd*, *ync*)^44^. This system is found in numerous SXT/R391 ICE elements and is generally also found inserted in HS2^15^. Examples of this system are displayed in Fig. 6 and compared with ICE*Pmi*Ire01 found in this study.

## Conclusion

Using a directed approach, a novel environmental SXT/R391 Type 1 family ICE, ICE*Pmi*Ire01 was isolated from a local municipal wastewater by using selective media for *Proteus* and *Vibrio* strains (common hosts for SXT/R391 ICEs) and monitoring the presence of ICEs using ICE specific *int* primers amplified by PCR. A *Proteus mirabilis* ICE*Pmi*Ire01 containing strain isolated in this way was subjected to genome sequencing and revealed an 81 kb element with 75 putative *orfs*. The element contained the 51 core ICE genes and contained insertions in some of its hotspots which indicated the presence of an encoded bacteriophage resistant mechanisms. A novel BREX system was identified in HS5, not previously identified or annotated in other SXT/R391 ICEs. Comparative analysis revealed that this system is also present in ICER391 but was not previously annotated there^40^ and in ICE*Vpa*Can1 again not previously annotated^7^. Unusually the nearest BREX homolog to ICE*Pmi*Ire01was reported in a *Bacillus subtilis* isolate which given the differences between Gram-positive and Gram-negative hosts is surprising. ICE*Pmi*Ire01 is a novel SXT/R391 ICE-like element with interestingly no antibiotic or metal resistance, the first to be identified in Ireland.

## Supplementary information

Supplementary information.
