# Supplementary material for: Isolation, nucleotide sequencing and genomic comparison of a Novel SXT/R391 ICE mobile genetic element isolated from a municipal wastewater environment
Source: Sci Rep. 2020 May 26;10:8716. doi: 10.1038/s41598-020-65216-5 (PMC7251087; doi:10.1038/s41598-020-65216-5)
Supplement: Supplementary file 1 — Supplementary information. [file 41598_2020_65216_MOESM1_ESM.docx]

**Isolation, nucleotide sequencing and genomic comparison of a Novel SXT/R391 ICE mobile genetic element isolated from a municipal wastewater environment**

**Shannon Slattery ^1^ J Tony Pembroke ^1,^ John G. Murnane^2^ and Michael P Ryan^1*^**

^1^ Department of Chemical Sciences, School of Natural Sciences, Bernal Institute, University of Limerick, Limerick, Ireland, V94 T9PX.

^2^ School of Engineering, University of Limerick, Limerick, Ireland, V94 T9PX.

***** Correspondence: [michaelpryan1983@gmail.com](mailto:michaelpryan1983@gmail.com)

**Supplementary Materials**

**Replica plating**

**1: Master Plate 2: Replica plate 1 3. Replica plate 2
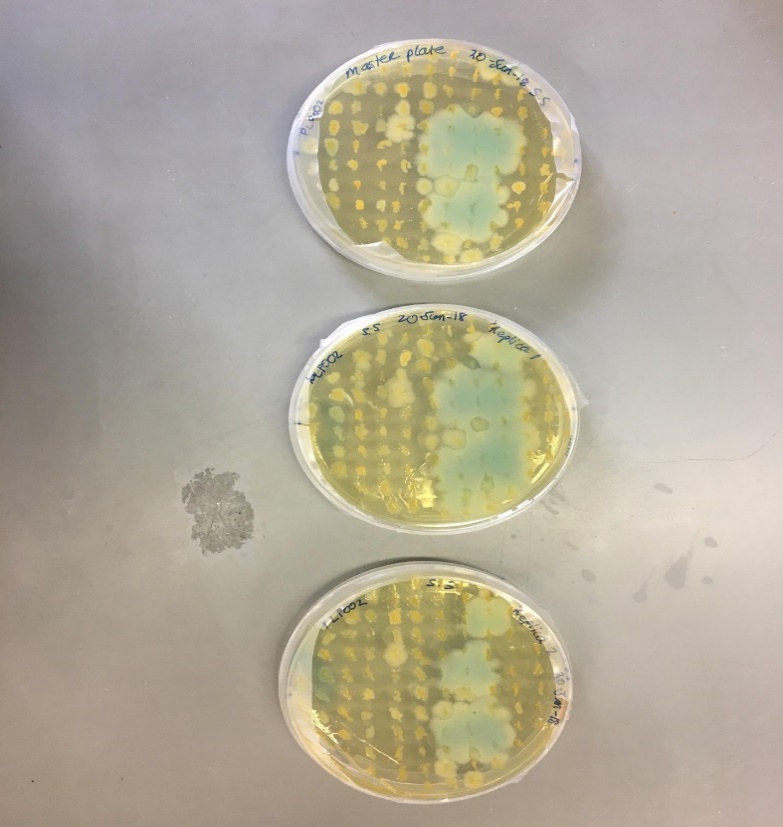
**

**Supplementary Figure 1:** Replica plating, of potential Proteus species containing an ICE mobile element on CLED media. Selected single colony and inoculated replicas using autoclaved toothpicks. Incubated for 24 hours at 37°C. Screened by taking a swab of one quarter/one half of the plate using a sterile cotton swab. If detected the area in question was replated and process undergone again until a single pure colony was isolated.

**Seasonal analysis**

Blue translucent colonies indicate the presence of a *Proteus* species

**
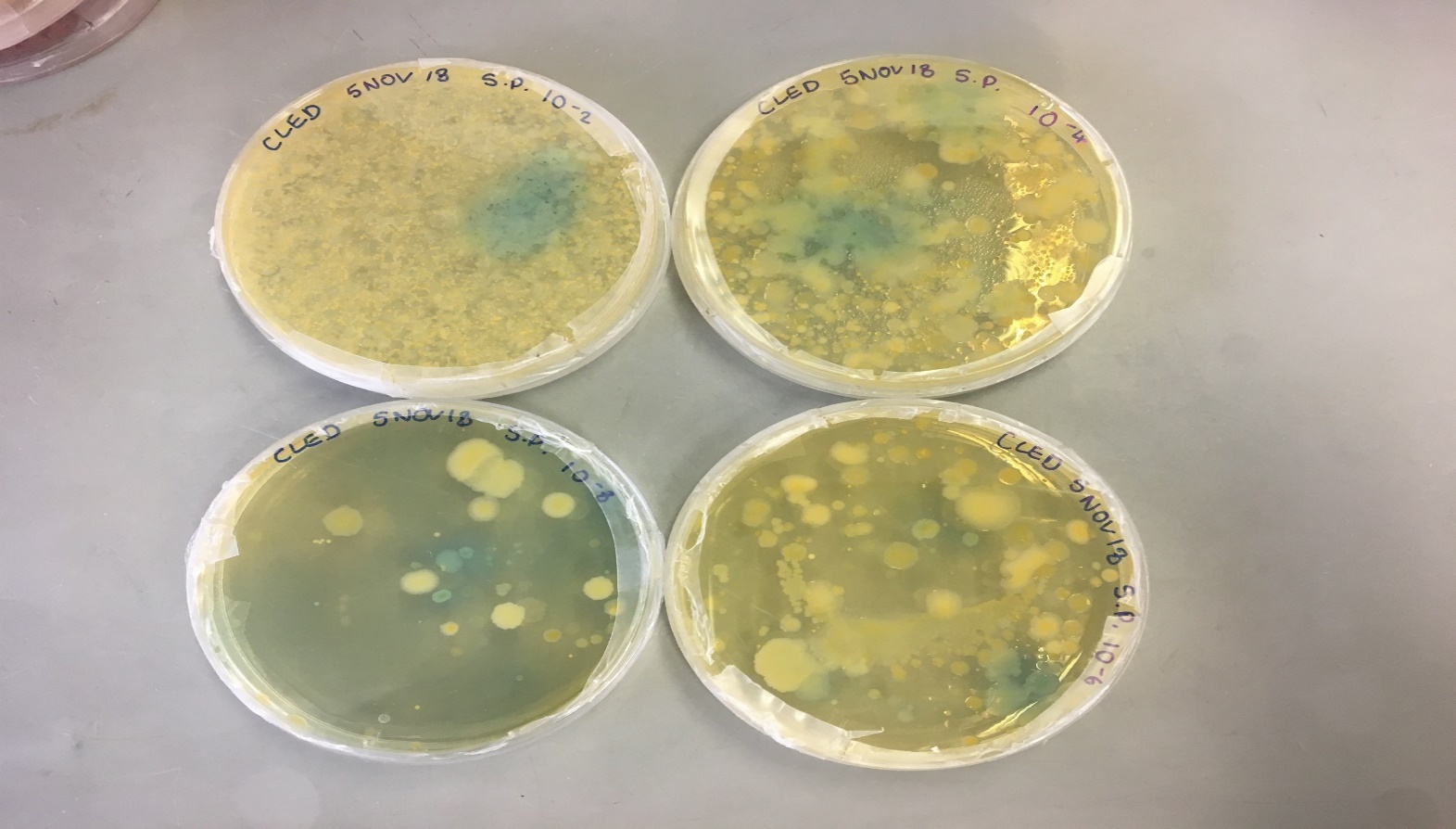
**

**Supplementary Figure 2:** Winter isolate at a dilution of 10^-2^ spread on CLED agar, translucent blue colonies indicate the presence of Proteus species (indicated by the red arrow) in a small portion of the plate and yellow colonies indicate the presence of E. coli, which cover the rest of the plate.

**M 1 2 3 4 5 6 7 8**


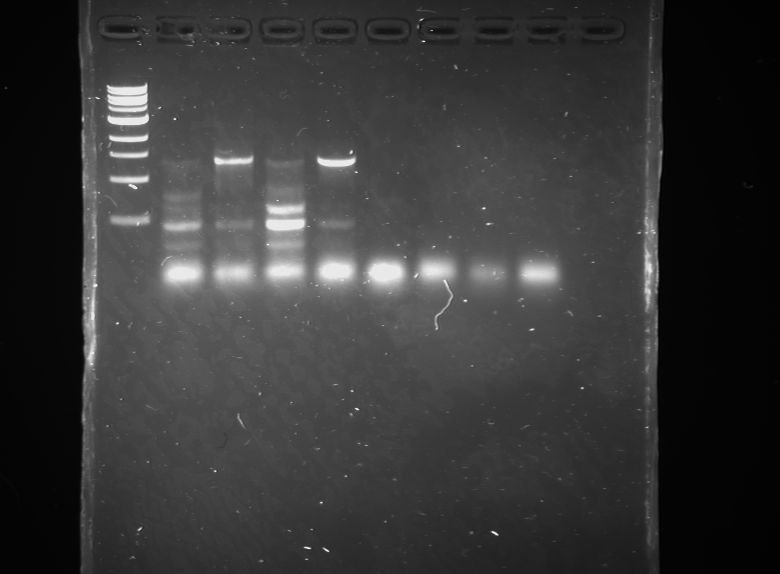


**Supplementary Figure 3:** Detection of integrase gene at 1378 bp from winter isolates on CLED media. **M**: 1 Kb Molecular weight maker, **1**: CLED 10^-2^ (Q1-rep1), **2:** CLED 10^-2^ (Q1-rep2), **3:** CLED 10^-2^ (Q2- rep1), **4:** CLED 10^-2^ (Q2-rep2), **5:** CLED 10^-2^ (repeat), **6:** CLED 10^-2^  (repeat), **7:** CLED 10^-4^ (1), **8:** CLED 10^-4^ (2). Electrophoresis carried out on a 1.2 % agarose gel stained with 5µl SYBR Safe stain (Edvotek), ran for 1 hour at 100V and photographed under UV.

**Full sequence analysis**

**Supplementary Table 1:** ORFs associated with ICE*Pmi*Ire01 isolated from *Proteus mirabilis* with percentage similarity to the prototype ICER391 and ICESXT with equivalent orfs and putative role of the encoded protein.

| ICE*Pmi*Ire01 | Percentage  similarity to  ICER391 | ICER391  equivalent | Percentage similarity  to  ICESXT | ICESXT equivalent | Putative role |
| --- | --- | --- | --- | --- | --- |
| orf01 | 100 % | orf02 (AAM08068.1) | N/A | N/A | Transcriptional regulator, Xre family |
| orf02 | 100 % | orf03 (AAM08010.1) | N/A | N/A | Serine/threonine-protein kinase HipA |
| orf03 | 100 % | orf04 (AAM08069.1) | N/A | N/A | DNA-binding protein |
| orf04 | 100 % | orf05 (AAM08088.1) | 100 % | Int (AAL59748.1) | Integration |
| orf05 | 100 % | orf06 (AAM08070.1) | 100 % | s002 (AAL59745.1) | Plasmid like protein |
| orf06 | 10 0% | orf07  (AAM08024.1) | 100 % | s003 (AAL59744.1) | Rod shape determination protein |
| orf07 | N/A | N/A | N/A | N/A | Hypothetical protein |
| orf08 | N/A | N/A | N/A | N/A | Hypothetical protein |
| orf09 | N/A | N/A | N/A | N/A | Origin of transfer |
| orf10 | 100% | RumB (AAM08023.1) | 100 % | RumB (AAL59754.1) | UV repair DNA polymerase |
| orf11 | 100% | RumA (AAM08055.1) | 97 % | RumA (AAL59746.1) | UV repair protein |
| orf12 | 100% | orf13 (AAM08020.1) | 100 % | s024 (AAL59738.1) | Exonuclease family protein |
| orf13 | 100% | orf14 (AAM08074.1) | 97 % | s025 (AAL59737.1) | Unknown function |
| orf14 | 97% | Hypothetical protein (AAM08019.1) | 97 % | s026 (AAL59736.1) | Unknown function |
| orf15 | 100% | orf23 (AAM08040.1) | N/A | N/A | DUF1819 family protein, Inner membrane protein |
| orf16 | 100% | orf24 (AAM08046.1) | N/A | N/A | DUF1788 domain-containing protein |
| orf17 | 100% | orf25 (AAM07998.1) | N/A | N/A | BREX system P-loop protein, BrxC |
| orf18 | N/A | N/A | N/A | N/A | Hypothetical Protein |
| orf19 | 100% | orf26 (AAM08051.1) | N/A | N/A | BREX-1 system adenine-specific DNA methyltransferase, PglX |
| orf20 | 93% | orf30 (AAM07999.1) | N/A | N/A | BREX-1 system phosphatase PglZ |
| orf21 | 97% | orf31 (AAM08002.1) | N/A | N/A | Protease Lon-related BREX system protein BrxL |
| orf22 | 100% | orf32 (AAM08000.1) | N/A | N/A | DNA repair protein |
| orf23 | 100% | TraI (AAM08003.1) | 93 % | TraI (AAL59675.1) | Conjugative relaxase |
| orf24 | 97% | TraD (AAM08004.1) | 97 % | TraD (AAL59680.1) | Conjugative coupling factor |
| orf25 | 95% | orf35 (AAM08047.1) | N/A | N/A | Conjugative transfer protein 234 |
| orf26 | 100% | orf36 (AAM08039.1) | 100 % | s043  (AAL59721.1) | Conjugative transfer protein s043 |
| orf27 | 93% | orf37 (AAM08045.1) | N/A | N/A | Plasmid-related protein |
| orf28 | 95% | orf38 (AAM08075.1) | N/A | N/A | Hypothetical Protein |
| orf29 | 95% | TraL (AAM08076.1) | 97 % | TraL (AAL59674.1) | Sex pilus assembly protein |
| orf30 | N/A | N/A | 97 % | TraE (AAL59679.1) | Sex pilus assembly |
| orf31 | 97% | TraK (AAM08021.1) | 97 % | s048 (AAL59718.1) | Sex pilus assembly protein |
| orf32 | 100% | TraB (AAM08009.1) | 100 % | TraB (AAL59682.1) | Sex pilus assembly protein |
| orf33 | 100% | HtdD (AAM08037.1) | 100 % | TraV (AAL59672.1) | Sex pilus assembly protein |
| orf34 | 97% | TraA (AAM08062.1) | 97 % | TraA (AAL59683.1) | Putative pilin subunit |
| orf35 | N/A | N/A | N/A | N/A | Transcriptional regulator, AbiEi 3 antitoxin |
| orf36 | N/A | N/A | N/A | N/A | Nucleotidyl AbiEii Toxin |
| orf37 | 100% | DsbC (AAM08034.1) | 100 % | s054 (AAL59715.1) | Putative disulfide bond isomerase |
| orf38 | 100% | TraC (AAM08001.1) | 100 % | TraC (AAL59681.1) | Sex pilus assembly protein |
| orf39 | N/A | N/A | N/A | N/A | Conjugative transfer protein |
| orf40 | 100 % | TrhF (AAM08054.1) | 97 % | TrsF (AAL59670.1) | Conjugation signal peptidase |
| orf41 | 88% | TraW (AAM08011.1) | 97 % | TraW (AAL59671.1) | Sex pilus assembly protein |
| orf42 | 100% | TraU (AAM08014.1) | 100 % | TraU (AAL59673.1) | Sex pilus assembly protein |
| orf43 | 100% | TraN (AAM07995.1) | N/A | N/A | Mating pair stabilisation protein |
| orf44 | N/A | N/A | N/A | N/A | Hypothetical protein |
| orf45 | N/A | N/A | N/A | N/A | Helicase HerA |
| orf46 | N/A | N/A | N/A | N/A | Endonuclease |
| orf47 | 100% | orf65 (AAM08043.1) | 100 % | s063 (AAL59711.1) | Plasmid-like protein |
| orf48 | 97% | orf66 (AAM08064.1) | N/A | N/A | Plasmid-like plasmid |
| orf49 | 100% | orf67 (AAM08058.1) | 100 % | ssb (AAL59691.1) | Single-stranded DNA binding protein |
| orf50 | 100% | orf68 (AAM08027.1) | 100 % | s065 (AAL59710.1) | Putative DNA recombination protein |
| orf51 | N/A | N/A | N/A | N/A | N/A |
| orf52 | 100% | orf69 (AAM08015.1) | 100 % | s066 (AAL59709.1) | Phage-type endonuclease domain protein |
| orf53 | 97% | orf70 (AAM08012.1) | 97 % | s067 (AAL59708.1) | Aerobic cobaltochelatase CobS subunit |
| orf54 | 97% | orf71 (AAM08029.1) | N/A | N/A | Unknown function |
| orf55 | 100 % | orf72 (AAM08016.1) | 100 % | s068 (AAL59707.1) | Cobalamin biosynthesis protein |
| orf56 | 95 % | orf73 (AAM08081.1) | 97 % | s069 (AAL59706.1) | Plasmid-like protein |
| orf57 | 100 % | orf74 (AAM08006.1) | 100 % | s070 (AAL59705.1) | Plasmid-like protein |
| orf58 | 97 % | orf75 (AAM08053.1) | 97 % | s071 (AAL59704.1) | Putative DNA repair protein |
| orf59 | 100 % | orf76 (AAM08083.1) | N/A | N/A | Plasmid-like protein |
| orf60 | 100 % | orf78 (AAM08013.1) | 100 % | s072 (AAL59703.1) | Primase |
| orf61 | 97% | orf79 (AAM08033.1) | 93 % | s073 (AAL59702.1) | Unknown function |
| orf62 | N/A | N/A | N/A | N/A | ATP binding |
| orf63 | N/A | N/A | N/A | N/A | 5-methylcytosine restriction system component |
| orf64 | 93% | TraF (AAM08018.1) | 97 % | TraF (AAL59678.1) | Sex pilus assembly protein |
| orf65 | 93% | TraH (AAM08008.1) | 95 % | TraH (AAL59676.1) | Sex pilus assembly protein |
| orf66 | 100% | TraG (AAM07996.1) | 100 % | TraG (AAL59677.1) | Sex pilus assembly protein |
| orf67 | 90 % | orf89 (AAM08057.1) | 97 % | s079 (AAL59699.1) | Eex |
| orf68 | 100 % | orf90 (AAM08050.1) | 97 % | SetC (AAL59693.1) | Transcriptional regulator |
| orf69 | 100 % | orf91 (AAM08085.1) | 100 % | SetD (AAL67891.1) | Transcriptional regulator |
| orf70 | 100 % | orf92 (AAM08048.1) | 95 % | s082 (AAL59698.1) | Soluble lytic murein transglycosylase |
| orf71 | 100 % | orf93 (AAM08041.1) | 100 % | s084 (AAL59696.1) | Unknown protein |
| orf72 | 100 % | orf94 (AAM08022.1) | N/A | N/A | Unknown protein |
| orf73 | N/A | N/A | 100 % | s086 (AAL59694.1) | Cro/Cl family transcriptional regulator |
| orf74 | 100% | orf96 (AAM08038.1) | 100 % | SetR (AAL59692.1) | Transcriptional repressor |
| orf75 | N/A | N/A | N/A | N/A | Peptide chain release factor 3 |

**Characterization and phenotypic testing**


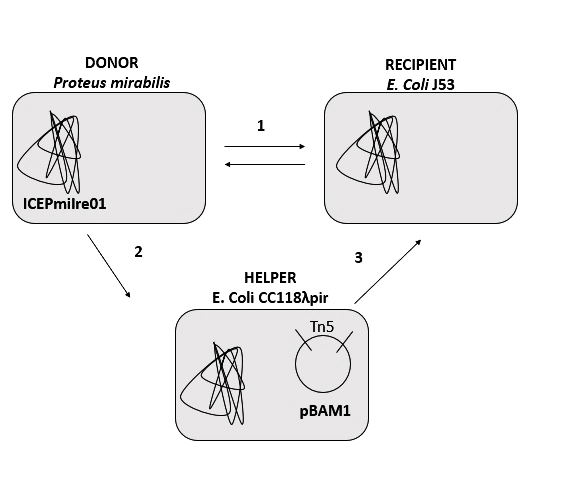


**Controls**

- Integrase positive (int +)
- Selective markers, Rifampicin and kanamycin

**Supplementary Figure 4**: Experimental design for Triparental mating of *Proteus mirabilis* containing ICE*Pmi*Ire01 (Host), *E. coli* CC118λpir containing pBAM1 with a Tn5 transposon (helper) and *E. coli* J53 rif, a rifampicin-resistant strain (Recipient). Triparental mating was carried out in a proportion of 5:1:1 (donor: helper: recipient).


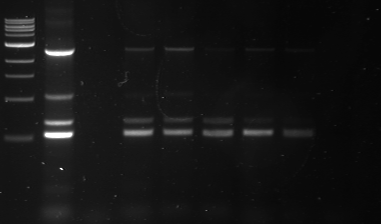


**Supplementary Figure 5**: PCR detection of *E. coli* using 16S rDNA primers. 1: Isolate 1, 2: Isolate 2, 3: Isolate 3, 4: Isolate 4, 5: Isolate 5, 6: Isolate 6, 7: Isolate 7, 8: Negative control (dH_2_O as template). Electrophoresis carried out on a 1.2 % agarose gel stained with 5µl SYBR Safe stain (Edvotek), ran for 1 hour at 100V and photographed under UV.


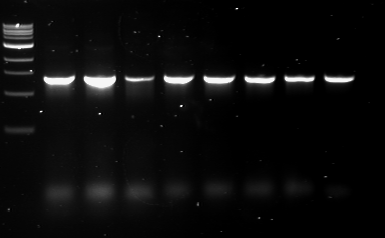


**Supplementary Figure 6:** PCR detection of the integrase gene using IntFor1 and IntRev1 primers, 1: Isolate 1, 2: Isolate 2, 3: Isolate 3, 4: Isolate 4, 5: Isolate 5, 6: Isolate 6, 7: Isolate 7, 8: Positive control (AB1157ICER391 as template), 9: Negative control (dH_2_O as template). Electrophoresis carried out on a 1.2 % agarose gel stained with 5µl SYBR Safe stain (Edvotek), ran for 1 hour at 100V and photographed under UV.

**Antibiotic screening**

**Supplementary Table 2:** List of antibiotics tested on the host *Proteus mirabilis* containing the ICE*Pmi*Ire01. Results determined by reference to the EUCAST Clinical Breakpoint tables for Enterobacterales and the CLSI zone diameter tables for Enterobacteriaceae.

| **Antibiotic** | ***Proteus mirabilis* (ICE*Pmi*Ire01)**  **Zone diameter (mm)** | **Result** |
| --- | --- | --- |
| **Penicillin’s** | | |
| Amoxycillin/clavulanic acid (20/10 µg) | 25 | Susceptible |
| Penicillin G (10 µg) | 20 | Susceptible |
| Ampicillin (10 µg) | 21 | Susceptible |
| Piperacillin (36 µg) | N/A | N/A |
| Piperacillin/tazobactam 30/6 µg) | 30 | Susceptible |
| Ticarcillin/clavulanic acid, 75/10 µg) | N/A | N/A |
| **Cephalosporins** | | |
| Cefoxitin (30 µg) | 20 | Susceptible |
| Cefpodoxime (10 µg) | 31 | Susceptible |
| Ceftazidime (10 µg) | 30 | Susceptible |
| Ceftiofur (30 µg) | 30 | Susceptible |
| Cefaclor (30 µg)) | 27 | Susceptible |
| Cephalothin (30 µg) | 20 | N/A |
| **Carbapenems** | | |
| Meropenem (10 µg) | 25 | Susceptible |
| **Monobactams** | | |
| Aztreonam (30 µg) | 35 | Susceptible |
| **Fluoroquinolones** |  |  |
| Norfloxacin (10 µg) | 20 | Susceptible |
| Ofloxacin (5 µg) | 18 | Resistant |
| **Aminoglycosides** | | |
| Amikacin (30 µg) | N/A | Resistant |
| Streptomycin (10 µg) | N/A | Resistant |
| Gentamicin (10 µg) | N/A | Resistant |
| Erythromycin (15 µg) | N/A | Resistant |
| Kanamycin (30 µg) | N/A | Resistant |
| Neomycin (30 µg) | 15 | Susceptible |
| **Tetracyclines** | | |
| Tetracycline (30 µg) | N/A | Resistant |
| Minocycline (30 µg) | 20 | Susceptible |
| **Miscellaneous agents** | | |
| SXT (1.25/23.75 µg) | N/A | Resistant |
| Trimethoprim (5 µg) | N/A | Resistant |

**Integron sequence**

A partial sequence of an Integron was found associated with NODE_40 on the host *Proteus mirabilis* sequence by searching bioinformatically for antibiotic resistance genes and then observing what genes resided upstream of the antibiotic resistance genes. Initially, a putative streptomycin 3''-O-adenylyltransferase gene and a linked integron integrase gene was located from 71800 to 72600 bp. This sequence and areas spanning this sequence was inputted into RAST and a molecular map was constructed using SnapGene, see supplementary Figure 5.

Integrons are defined by the presence of an integron integrase gene and a proximal primary recombination site (attL). The different amino acid sequences of the integron integrases are how the different classes are classified with 4 major types (Deng *et al.* 2015). The *Proteus* chromosomal integron was classified bioinformatically as a class II integron which contains a gene cassette that contains genes that confer resistance to lincomycin, trimethoprim and streptomycin. Classic class 2 integrons contain an array of gene cassettes including dihydrofolate reductase, streptomycin acetyltransferase and aminoglycoside acetyltransferase which confers resistance to trimethoprim, streptothricin and streptomycin (Deng *et al.* 2015). This integron sequence gives the host organism, in this case, *Proteus mirabilis* antibiotic resistance which provides the host with an adaptive advantage.


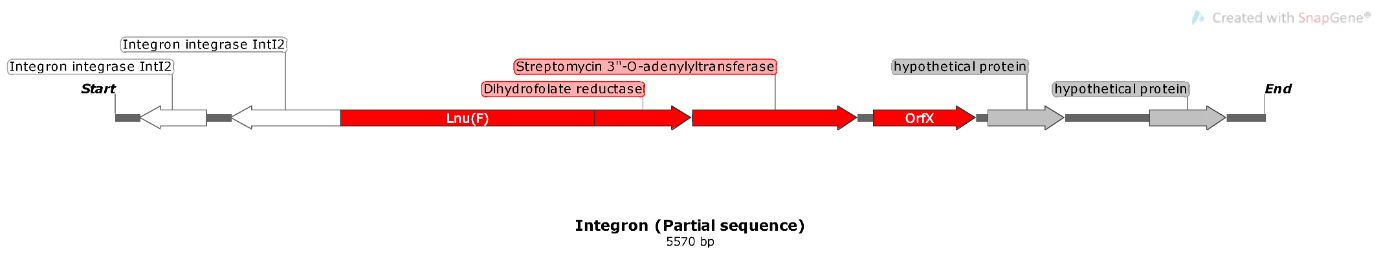


**Supplementary Figure 7:** Molecular map of partial sequence from a Class 2 integron isolated from a Proteus mirabilisICEPmiIre01 isolate. This partial sequence contains eight orfs and is 5570 bp in size.

**Supplementary Table 3:** Efflux pumps identified in the host chromosome of *P. mirabilis*. Obtained based on the full sequence data of the host, labelled by the name, location, size and putative role.

| **Name** | **Location** | **Size (aa)** | **Putative role** |
| --- | --- | --- | --- |
| *eamA* | 5175..6080 | 301 | O-acetylserine/cysteine exporter |
| *arpC* | 9330..9818 | 162 | Efflux transporter outer membrane subunit |
| *acrZ_2* | 107..250 | 47 | Multidrug efflux pump-associated protein, AcrZ family |
| *srpA_2* | 29874..31013 | 379 | Efflux RND transporter periplasmic adaptor subunit |
| *mntP_2* | 5981..6565 | 194 | Manganese efflux pump MntP |
| *acrA_1* | 48052..49242 | 396 | Efflux RND transporter periplasmic adaptor subunit |
| *bepE_1* | 247392..250469 | 1025 | MexW/MexI family multidrug efflux RND transporter permease subunit [*Proteus mirabilis*] |
| *fieF_1* | 22284..23165 | 293 | Cation diffusion facilitator transporter family protein [ *Proteus mirabilis*] |
| *acrB_2* | 44879..48037 | 1052 | Multidrug efflux RND transporter permease subunit [*Proteus mirabilis*] |

**Data contained in the phylogenetic tree**

**Supplementary Table 4:** Identified ICE_SXT/R391_ family members with a complete genome sequence.

SXT/R391 ICEs with ** are not annotated or partially annotated found in the study by Bioteau et al. (2018), these ICEs are scattered over two contigs with extracted WGS data, an estimation of the minimum size is provided.

*****Unpublished Sequence data ****** Not annotated or partially annotated

| **Element** | **Strain** | **Environment** | **Location/Year of isolation** | **Accessory Genes/Reported Functions** | **Acc. No.** | **Size (kb)** | **Ref** |
| --- | --- | --- | --- | --- | --- | --- | --- |
| ICE R391 | *Providencia rettgeri* | Clinical | Pretoria, South Africa/1967 | Km^r^, Hg^r^, DNA repair genes, sulphate transporter, toxin-antitoxin system, ATP-dependent Lon protease | AY090559 | 89 | (Böltner *et al.* 2002) |
| ICEpMERPH | *Shewanella putrefaciens* | River isolate | United Kingdom/1987 | Hg^r^, As^r^ | MH974755 | 110 | This study |
| ICE*Pmi*Ire01 | *Proteus mirabilis* | Wastewater | Ireland/2018 | BREX (bacteriophage exclusion) system, RMS, abi system, toxin-antitoxin system | MN520463 | 81 | This study |
| ICESXT (MO10) | *Vibrio cholerae* O139 MO10 | Clinical | India/1992 | Cm^r^, Su^r^, Tm^r^, Spt^r^, DNA repair genes, toxin-antitoxin system, ATP-dependent Lon protease, histidine kinase, diguanylate cyclase, deoxycytidine triphosphate deaminase | AY055428 | 99 | (Beaber *et al.* 2002) |
| ICER997 | *Proteus mirabilis* | Clinical | Indian sub-continent/1978 | Β-lactamase gene, DNA repair genes, RM system, toxin-antitoxin system | KY433363 | 85 | (Ryan *et al.* 2017) |
| ICE*Ama*MED64 | *Alteromonas macleodii* 'Aegean Sea MED64' | Superficial waters | Lebanon, Aegean Sea/2000 | type I RMS | [CP004848](https://www.ncbi.nlm.nih.gov/nuccore/CP004848)  (1194615-1274449) | 80 | (López-Pérez *et al.* 2013) |
| ICE*Ama*IS1 | *Alteromonas macleodii* Ionian Sea UM7 | Marine | Ionian Sea, Uranian Basin west of Crete, Greece/2012 | Heavy metal resistance, type I RMS | NC_021713 | 99 | (López-Pérez *et al.* 2013) |
| ICE*Mfu*Ind1a | *Marinomonas fungiae* JCM 18476 | Mucus of the coral *Fungia echinata* | Andaman Sea, India/2010 | RMS | LIQF00000000 | 66 | (Badhai and Das 2016) |
| ICE*Mfu*Ind1b | *Marinomonas fungiae* JCM 18476 | Mucus of the coral *Fungia echinata* | Andaman Sea, India/2010 | RMS | LIQF00000000 | 75 | (Badhai and Das 2016) |
| ICE*Mpr*Chn1 | *Marinomonas profundimaris* D104 | Surface sediment | Arctic Ocean/2010 | RMS | AYOZ01000000 | 86 | (Dong *et al.* 2014) |
| ICE*Pal*Ban1 | *Providencia alcalifaciens* | Clinical | Bangladesh/1999 | Cm^r^, Spt^r^, Su^r^, Tm^r^, toxin-antitoxin system, phenazine biosynthesis protein, lysine exporter, glyoxalase resistance, RMS | GQ463139 | 97 | (Wozniak *et al.* 2009) |
| ICE*Pda*Spa1 | *Photobacterium damselae*  subsp*. piscicida* PC554.2 | Marine | Spain/2001 | Tc^r^, heat-shock protein (dnaK), AAA ATPase, toxin-antitoxin system, ATP-dependent Lon protease ICE*Pmi*USA1 | AJ870986 | 103 | (Osorio *et al.* 2008) |
| ICE*Pmi*Jpn1 | *Proteus mirabilis* | Clinical | Japan/2006 | Amx^r^, Amc^r^, Fox^r^, Ctx^r^ | KT894734 | 93 | (Lei *et al.* 2016) |
| ICE*Pmi*USA1 | *Proteus mirabilis*  *strain* HI4320 | Clinical | USA | ATP-dependent helicase, DNA repair proteins, toxin-antitoxin system | AM942759 | 81 | (Pearson *et al.* 2008) |
| ICE*Spu*PO1 | *Shewanella putrefaciens* W3-18-1 | Marine sediment | Pacific Ocean/2000 | Cu^r^, Zn/Co/Cd RND efflux pump, DNA repair genes, restriction modification system | CP000503 | 111 | (Pembroke and Piterina 2006) |
| ICE*Va*lA056-1 | *Vibrio alginolyticus* A056 | Gill of a Litopenaeus vannamei | China/2003 | Spt^r^, Su^r^, Az^r^, type III RM system | KR231688 | 89 | (Luo *et al.* 2016) |
| ICE*Val*A056-2 | *Vibrio alginolyticus* A056 | Gill of a Litopenaeus vannamei | China/2003 | type I RM system | KR231689 | 104 | (Luo *et al.* 2016) |
| ICE*Val*ASI1* | *Vibrio alginolyticus* ANC4-19 | Marine | Andaman and Nicobar Islands, India/2010 | L-lactate degradation system | NZ_LTYK00000000 | N/A | (Bhotra and Singh 2016) |
| ICE*Val*E0601 | *Vibrio alginolyticus* E0601 | Marine (Seawater) | China/2006 | type III RM system | KT072768 | 106 | (Luo *et al.* 2016) |
| ICE*Val*HN396 | *Vibrio alginolyticus* HN396 | Marine (Seawater) | China/2008 | type II RMS | KT072770 | 87 | (Luo *et al.* 2016) |
| ICE*Val*HN437 | *Vibrio alginolyticus* HN437 | Marine  (Seawater) | China/2008 | type I RM system | KT072771 | 94 | (Luo *et al.* 2016) |
| ICE*Val*HN492 | *Vibrio alginolyticus* HN492 | Marine (Seawater) | China/2008 | type III RM system | KT072769 | 106 | (Luo *et al.* 2016) |
| ICE*Vch*Ban11 | *Vibrio cholerae* 4672 | Clinical | Bangladesh/2000 | type II restriction system | ERS016137 | 96 | Direct submission |
| ICE*Vch*Ban5 | *Vibrio cholerae* O1 | Clinical | Bangladesh/1998 | Cm^r^, Spt^r^, Su^r^, Tm^r^, toxin-antitoxin system | GQ463140 | 102 | (Wozniak *et al.* 2009) |
| ICE*Vch*Ban8 | *Vibrio cholerae*  O37 strain MZ03 | Clinical | Bangladesh/2001 | Ac^r^, Toxin-antitoxin system | JQ345361 | 103 | (Wozniak *et al.* 2009) |
| ICE*Vch*Ban9 | *Vibrio cholerae* MJ-1236 | Clinical | Bangladesh/1994 | Cm^r^, Spt^r^, Su^r^, Tm^r^, Tc^r^ | CP001485 | 108 | (Wozniak *et al.* 2009) |
| ICE*Vch*Chn0143 | *Vibrio cholerae* ICDC-VC0143 | Clinical | China/2001 | Spt^r^, Su^r^, Tm^r^, Tc^r^, toxin-antitoxin system | KT151654 | 87 | (Wang *et al.* 2016) |
| ICE*Vch*Chn0956 | *Vibrio cholerae* ICDC-VC956 | Water | China/2001 | Spt^r^, Su^r^, Tm^r^, toxin-antitoxin system | KT151655 | 94 | (Wang *et al.* 2016) |
| ICE*Vch*Chn1605 | *Vibrio cholerae* ICDC-VC1605 | Clinical | China/1993 | Spt^r^, Su^r^, Tm^r^, toxin-antitoxin system | KT151656 | 98 | (Wang *et al.* 2016) |
| ICE*Vch*Chn1627 | *Vibrio cholerae* ICDC-VC1627 | Clinical | China/1997 | Spt^r^, Su^r^, Tm^r^, Tc^r^, toxin-antitoxin system | KT151657 | 102 | (Wang *et al.* 2016) |
| ICE*Vch*Chn1909 | *Vibrio cholerae* ICDC-VC1909 | Clinical | China/1998 | Spt^r^, Su^r^, Tm^r^, toxin-antitoxin system | KT151658 | 109 | (Wang *et al.* 2016) |
| ICE*Vch*Chn1944 | *Vibrio cholerae* ICDC-VC1944 | Unknown | China/2000 | Spt^r^, Su^r^, Tm^r^, toxin-antitoxin system | KT151659 | 101 | (Wang *et al.* 2016) |
| ICE*Vch*Chn2255 | *Vibrio cholerae* ICDC-VC2255 | Clinical | China/2008 | Spt^r^, Su^r^, Tm^r^, Tc^r^ ,type I RM system, toxin-antitoxin system | KT151660 | 95 | (Wang *et al.* 2016) |
| ICE*Vch*Chn2605 | *Vibrio cholerae* ICDC-VC2605 | Clinical | China/1998 | Spt^r^, Su^r^, Tm^r^, toxin-antitoxin system | KT151661 | 98 | (Wang *et al.* 2016) |
| ICE*Vch*Chn*306** | *Vibrio cholerae* E306 | Clinical | China/2013 | toxin-antitoxin system, ATP-dependent Lon protease | NZ_AWWA01000000 | N/A | (Yi *et al.* 2014) |
| ICE*Vch*Chn4210 | *Vibrio cholerae* ICDC-VC4210 | Clinical | China/1999 | Spt^r^, Su^r^, Tm^r^, Tc^r^, toxin-antitoxin system | KT151662 | 110 | (Wang *et al.* 2016) |
| ICE*Vch*Chn57 | *Vibrio cholerae* ICDC-VC57 | Clinical | China/2005 | Spt^r^, Su^r^, Tm^r^, Tc^r^, toxin-antitoxin system | KT151664 | 96 | (Wang *et al.* 2016) |
| ICE*Vch*Chn1307 | *Vibrio cholerae* ICDC-1307 | Clinical | China | Cm^r^, Tm^r,^ DNA repair genes | KJ817376.1 | 104 | Direct submission |
| ICE*Vch*ChnAHV1003 | *Vibrio cholerae* AHV1003 | Clinical | China/2010 | Em^r^, Spt^r^, Su^r^, Tm^r^, type I RM system, toxin-antitoxin system | KT151663 | 102 | (Wang *et al.* 2016) |
| ICE*Vch*Hai1 | *Vibrio cholerae* VC1786 | Clinical | Haiti/2010 | Tm^r^, Su^r^, Spt^r^, toxin-antitoxin system | JN648379 | 98 | (Sjölund-Karlsson *et al.* 2011) |
| ICE*Vch*Hai2 | *Vibrio cholerae* HC-1A2 | Clinical | Haiti/2010 | RM system | AJRO01000008 | 84 | (Ceccarelli *et al.* 2013) |
| ICE*Vch*Ind4 | *Vibrio cholerae* 0139 | Clinical | India/1997 | Cm^r^, Sptr, Su^r^, Toxin-antitoxin system | GQ463141 | 95 | (Wozniak *et al.* 2009) |
| ICE*Vch*Ind5 | *Vibrio cholerae* O1 | Clinical | India/1994 | Cm^r^, Spt^r^, Su^r^, Tm^r^, glyoxalase resistance, toxin-antitoxin system | GQ463142 | 98 | (Wozniak *et al.* 2009) |
| ICE*Vch*Ind6 | *Vibrio cholerae* 4605 | Clinical | India/2007 | Tm^r^ | ERS013257 | 90 | (Chowdhury *et al.* 2015) |
| ICE*Vch*Mex1 | *Vibrio cholerae*  non O1-0139 | Sewage sample | Mexico/2001 | RMS, toxin-antitoxin system | GQ463143 | 83 | (Wozniak *et al.* 2009) |
| ICE*Vch*Moz10 | *Vibrio cholerae* O1 El Tor B33 | Clinical | Mozambique/2004 | Spt^r^, Su^r^, Tc^r^, toxin-antitoxin system | ACHZ00000000 | 104 | (Taviani *et al.* 2009) |
| ICE*Vch*B33 | *Vibrio cholerae* O1 El Tor MJ123 | Clinical | India/1994 | Tm^r^, Cm^r^, Tc^r^, Su^r^, Sp^r^, Co:Zn:Cd resistance | ACHZ00000000 | 106 | (Taviani *et al.* 2009) |
| ICE*Vfl*Bra1* | *Vibrio fluvialis*560 | Marine (Oyster) | Brazil/2002 | RMS | JQHX00000000 | N/A | (de Oliveira Veras *et al.* 2015) |
| ICE*Vfl*Bra2* | *Vibrio fluvialis*539 | Marine (Oyster) | Brazil/2002 | RMS | JQHW00000000 | N/A | (de Oliveira Veras *et al.* 2015) |
| ICE*Vfl*Ind1 | *Vibrio fluvialis* | Clinical | India/2002 | Cm^r^, Sm^r^, Su^r^, Tm^r^, toxin-antitoxin system, RMS | GQ463144 | 114 | (Wozniak *et al.* 2009) |
| ICE*Apl*2 | *Actinobacillus pleuropneumoniae MIDG3553* | Clinical | United Kingdom/2012 | Sm^r^, Su^r^, Tm^r^, DNA repair genes, RMS | MF187965.1 | 94 | (Li *et al.* 2017) |
| ICE*Vch*Thd1 | *Vibrio Cholerae* TSY216 | Clinical | Thailand/2010 | Cm^r^, Su^r^, Tc^r^, Tm^r^, Ma^r^ and RMS | CP007653.1  (579719-680801) | 101 | (Okada *et al.* 2015) |
| ICE*VFlH*-08942 | *Vibrio fluvialis* H08942 | Clinical | India/2002 | Sm^r^, Cm^r^, Tm^r^, DNA repair genes, RMS | KM213605.1 | 103 | (Poulin-Laprade *et al.* 2015) |
| ICE*Elba*HL53 | *Idiomarinaceae bacterium,* HL-53 | Unknown | Unknown | RMS | LN899469.1 | 69 | Direct submission |
| ICE*Alt*Mex1 | *Alteromonas sp.* Mex14 | Aquaculture pond | Mexico/2014 | RMS, Heavy metal resistance protein | CP018023 | 83 | (López-Pérez *et al.* 2017) |
| ICE*PSt*33672 | *Providencia stuartii* ATCC 33672 | Unknown | Unknown | RMS, Hg^r^ | CP008920.1 | 76 | (Frey *et al.* 2014) |
| ICE*Val*Chn1 | *Vibrio alginolyticus* ZJ-T | Marine (Epinephelus coioides) | China/2005 | Fq^r^, Tm^r^, Su^r^, Ag^r^, RMS | CP016224.1 | 86 | Direct submission |
| ICE*Vch*Rua1 | *Vibrio Cholerae* RND68789 | Clinical | Russia/2012 | Bm^r^, Spt^r^, Su^r^, DNA repair genes, abortive phage resistance protein | KY382507.1 | 98 | (Kuleshov *et al.* 2016) |
| ICE*Vch*Rua2 | *Vibrio cholerae O1 biovar El Tor* Inaba RND18826 | Clinical | Russia/2012 | Bm^r^, Spt^r^, Su , DNA repair genes, abortive phage resistance protein | KY382506.1 | 98 | (Kuleshov *et al.* 2016) |
| ICE*Vch*Nig1 | *Vibrio Cholerae* VC833 | Clinical | Nigeria/2010 | Spt^r^, Tm^r^ Bm^r^ Su^r^, Flo^r^ , DNA repair genes | KC886258.1 | 98 | (Marin *et al.* 2014) |
| ICE*Vch*Nep1 | *Vibrio Cholerae* VC504 | Clinical | Nepal/1994 | Spt^r^, Tm^r^ Bm^r^ Su^r^ , DNA repair genes | KC886257.1 | 98 | (Marin *et al.* 2014) |
| ICE*Pvu*CHN2213 | *Proteus vulgaris*  08MAS2213 | Food | China/2008 | Bm ^r^ DNA repair genes, RMS  Am^r^, Az^r^, Cm^r^, Cfz^r^, A/S^r^, SXT^r^, Su^r^, Sm^r^ | KX243403.1 | 94 | (Li *et al.* 2016) |
| ICE*Pmi*Chn1 | *Proteus mirabilis* PM13C04 | Poultry | China/2012-2014 | DNA repair genes, Flo^r^, Tc^r^, Spt^r^, Su^r^ | KT962845.1 | 94 | (Lei *et al.* 2016) |
| ICE*Pmi*Chn2 | *Proteus mirabilis* JN7 | Poultry | China/2013 | Su^r^, Bm^r^ ultraviolet light resistance protein B, DNA repair genes | KY437726.1 | 106 | (Bie *et al.* 2017) |
| ICE*Pmi*Chn3 | *Proteus mirabilis* JN28 | Poultry | China/2013 | Hm^r^, Tm^r^, Em^r^, Spt^r^, DNA repair genes | KY437727.1 | 57 | (Bie *et al.* 2017) |
| ICE*Pmi*Chn4 | *Proteus mirabilis* JN49 | Poultry | China/2013 | β-lactamase, DNA repair genes, Sum^r^, Spt^r^, Flo^r^ | KY437728.1 | 92 | (Bie *et al.* 2017) |
| ICE*Pmi*CHN1586 | *Proteus mirabilis* 08MAS1586 | Food | China/2008 | Tm^r^, Flo_r_, Bm^r^, Spt^r^, Su^r^, DNA repair genes | KX243404.1 | 99 | (Li *et al.* 2016) |
| ICE*Pmi*CHN2407 | *Proteus mirabilis* 09MAS2407 | Clinical | China/2009 | Tc^r^, RMS, Co-Zn-Cd resistance, DNA repair genes, Hg^r^ | KX243405.1 | 97 | (Li *et al.* 2016) |
| ICE*Pmi*CHN2410 | *Proteus mirabilis* 09MAS2410 | Clinical | China/2009 | DNA repair polymerases, RMS, Co-Zn-Cd resistance, Hg^r^, Am^r,^ Azm^r^, Cm^r^, Km^r^, Sptr, SXT^r^, Su^r^, Tc^r^, CFZ^r^, Cip^r^ | KX243406.1 | 93 | (Li *et al.* 2016) |
| ICE*Pmi*CHN2416 | *Proteus mirabilis* 09MAS2416 | Clinical | China/2009 | RMS, Co-Zn-Cd resistance, DNA repair genes, Hg^r^ | KX243407.1 | 92 | (Li *et al.* 2016) |
| ICE*Pmi*CHN901 | *Proteus mirabilis* MD20140901 | Clinical | China/2014 | Tm^r^, Bm^r^, Flo^r^, Spt^r^, RMS, DNA repair genes | KX243208.1 | 89 | (Li *et al.* 2016) |
| ICE*Pmi*CHN902 | *Proteus mirabilis* MD20140902 | Clinical | China/2014 | Bm^r^, Spt^r^, Su^r^, Am^r^ Azm^r^, Az^r^, Amk^r^, Cm^r^, Cip^r^, Km^r^ ,SXT^r^, Tc^t^ , DNA repair genes, RMS | KX243409.1 | 89 | (Li *et al.* 2016) |
| ICE*Pmi*CHN903 | *Proteus mirabilis MD20140903* | Clinical | China/2014 | Bm^r^, Tm^r^, Sptr, Spt^r^, RMS, DNA repair genes | KX243410.1 | 90 | (Li *et al.* 2016) |
| ICE*Pmi*CHN904 | *Proteus mirabilis*  MD20140904 | Clinical | China/2014 | Bm^r^, RMS, Co-Zn-Cd resistance, DNA repair genes, Spt^r^, Su^r^, Tc^r^ | KX243411.1 | 95 | (Li *et al.* 2016) |
| ICE*Pmi*CHN905 | *Proteus mirabilis* MD20140905 | Clinical | China/2014 | Bm^r^, RMS, Tc^r^, Spt^r^, Su^r^, Co-Zn-Cd resistance protein | KX243412.1 | 95 | (Li *et al.* 2016) |
| ICE*Pmi*CHN1809 | *Proteus mirabilis* TJ1809 | Clinical | China/2013 | Am^r^, Km^r^, SXT^r^, Azm^r^, Cm^r^, Su^r^, Tc^r^, Cf^r^ | KX243413.1 | 76 | (Li *et al.* 2016) |
| ICE*Pmi*CHN3237 | *Proteus mirabilis*  TJ3237 | Clinical | China/2013 | DNA repair genes, RMS, CFZ^r^ ,Am^r^, Azm^r^, SXT^r^, Tc^r^, Su^r^ | KX243414.1 | 87 | (Li *et al.* 2016) |
| ICE*Pmi*CHN3300 | *Proteus mirabilis* TJ3300 | Clinical | China/2013 | Tm^r^, Azm^r^, Cm^r^, SXT^r^, Su^r^, Spt^r^, DNA repair genes | KX243415.1 | 108 | (Li *et al.* 2016) |
| ICE*Pmi*CHN3335 | *Proteus mirabilis TJ3335* | Clinical | China/2013 | Azm^r^ , Bm^r^, RMS, Tc^r^, Spt^r^, DNA repair polymerases | KX243416.1 | 90 | (Li *et al.* 2016) |
| ICE*Pmi*Chn15C1 | *Proteus mirabilis Pm15C1* | Poultry | China | DNA repair genes, Tm^r^, RMS | KX268685.1 | 65 | Direct Submission |
| ICE*Vfl*Chn1* | *Vibrio fluvialis* 12605 | Clinical (bile) | China/2013 | Bile resistance genes (toxR, ompU, ompT, tolC), Heat stock proteins (GroES, GroEL, HspA), virulence factors & prophage regions | CP019118.1 | N/A | Direct Submission |
| ICE*Vflu*Chn2* | *Vibrio fluvialis* 12605 | Clinical (bile) | China/2013 | Bile resistance genes (toxR, ompU, ompT, tolC), Heat stock proteins (GroES, GroEL, HspA), virulence factors & prophage regions | CP019119.1 | N/A | Direct Submission |
| ICE*Pmi*AR_0159 | *Proteus mirabilis* AR_0159 | Unknown | Unknown | Cm^r^, Flo^r^, Tm^r^, Heavy metal resistance protein. | CP021550.1 | 99 | Direct Submission |
| ICE*Sup*CHN110003 | *Shewanella upenei* 110003 | Clinical | China/2011 | DNA repair genes, Bm^r^, Cm^r^, Spt^r^, Su^r^, RMS. | MG014393.1 | 92 | (Fang *et al.* 2018) |
| ICE*Pmi*Fra1 | *Proteus mirabilis* PmPHI | Clinical | France/2012 | Flo^r^, Cm^r^ Spt^r^, Su^r^, Tc^r^, Km^r ,^ Tm^r^, Em^r^, Nm^r^, RMS, merR family gene & DNA repair genes | MF490434.1 | 107 | (Siebor *et al.* 2018) |
| ICE*Sha*Jpn1* | *Shewanella halifaxiensis* 6JANF4-E-4 | Marine (fish intestine) | Japan (Ehime, Uwa Sea)/2013 | Ma^r^, Flo^r^, Su^r^, β-lactamase | BFBQ01000001.1 | N/A | (Sugimoto *et al.* 2018) |
| ICE*Sha*Jpn2* | *Shewanella halifaxiensis* 6JANF4-E-4 | Marine (fish intestine) | Japan (Ehime, Uwa Sea)/2013 | Ma^r^, Flo^r^, Su^r^, β-lactamase | BFBQ01000005.1 | 44.6 | (Sugimoto *et al.* 2018) |
| ICE*Apl*Chn1 | *Actinobacillus pleuropneumoniae* App6 | Animal (lung sample from a pig with respiratory disease) | China/2013 | Flo^r,^ Cm^r^, Tc^,^ Amk^r^, Km^r^, Spt^r^, Til^r^, Em^r^, Clr^r^, Lm^r^, Su^r^ | KX196444 | 100 | (Xu *et al.* 2018) |
| ICE*Pmi*ChnBCP11 | *Proteus mirabilis* BCP11 | Diarrheic piglet | China | Tc^r^, Km^r^, Nm^r^, Blm^r^, Rif^r^, Hym^r^, Am^r^, Spt^r^, Ctx^r^, Az^r^, Spm^r^, Ap^r^, To^r^, Tm^r^, SXT, Cf^r^, Nf^r^, Fm^r^, Cm^r^, Flo^r^, RMS, Nal^r^ | MG773277.1 | 141 | (Lei *et al.* 2018) |
| ICE*Asp*BS1 | *Alteromonas sp.* RW2A1 | Seawater | Baltic Sea/2013 | Calcium/sodium antiporter, RMS, multidrug efflux RND transporter, heavy metal transporter | CP018031.1  (1192908-2109146) | 116 | (López-Pérez *et al.* 2017) |
| ICE*Vpa*Can1 | *Vibrio parahaemolyticus* S107-1 | Marine (Oyster) | Canada/2005 | BREX (bacteriophage exclusion system), toxin HipA, XRE family transcriptional regulator | CP028481 | 79 | (Bioteau *et al.* 2018) |
| ICE*Sh*95 | *Shewanella sp. Sh95* | Clinical (ocular secretion) | Argentina/2005 | *blaOXA-48,* RMS, ArsR family transcriptional regulator, zinc transporter, multidrug transporter, heavy metal transport, MerR family transcriptional regulator, cobalt transporter, dihydrofolate reductase, ethidium bromide resistance. | LGYY01000082  LGYY01000240.1 | 110 | (Di Noto *et al.* 2016) |
| ICE*Ama*D7 | *Alteromonas macleodii, D7* | Marine (seawater) | Thailand (Andaman Sea)/2000 | Hg^r^, Ar^r^, Cu^r^, Ca proton antiporter, mechanosensitive ion channel, RMS, multidrug transporter, hydrophobe/amphiphile efflux protein, | CP014323  (1261745-1375908) | 114 | Direct submission |
| ICE*Eco*HVH177 | *Escherichia coli*  HVH 177 | Clinical | Denmark/2003 | Unknown | AZJM01000017 | 97 | Direct submission |
| ICE*Mfr*JAM7 | *Methylophaga frappieri* JAM7 | Marine (Seawater) | Canada/Unknown | RMS, toxin-antitoxin system, molybdenum cofactor biosynthesis enzyme, multidrug resistance efflux pump | CP003380  (495596-606053) | 110 | (Villeneuve *et al.* 2012) |
| ICE*Sde*ChnS12 | *Shewanella decolorationis* S12 | Wastewater treatment plant | China/2012 | AAA ATPase, RMS | AXZL01000060.1 | 71 | (Xu *et al.* 2013) |
| ICE*Val*ZJT1 | *Vibrio alginolyticus* ZJ-T | Marine (Orange-spotted grouper) | China/2005 | Fq^r^, Km^r^, Tm^r^, RMS | CP016224  (2476706-2564476) | 89 | (Deng *et al.* 2016) |
| ICE*Val*ZJT2 | *Vibrio alginolyticus* ZJ-T | Marine (Orange-spotted grouper) | China/2005 | RMS, Calcium: sodium antiporter | CP016224  (936462-1036489) | 100 | (Deng *et al.* 2016) |
| ICE*Vch*2012HC25** | *Vibrio cholerae* O1 El Tor  2012HC-25 | Clinical | Haiti/2012 | NA | JSTY01000047 | 77 | (Azarian *et al.* 2014) |
| ICE*Vch*2012Env25** | *Vibrio cholerae* non-O1/O139  2012Env-25 | Water | Haiti/2012 | NA | JSTE01000047 | 108 | (Azarian *et al.* 2014) |
| ICE*Vch*8-76-1** | *Vibrio cholerae* O77 8-76-1 | Clinical | India/1976 | NA | JIDN01000032 | 102 | (Bishop-Lilly *et al.* 2014) |
| ICE*Vch*8-76-2** | *Vibrio cholerae*  O77 8-76-2 | Clinical | India/1976 | NA | JIDN01000012 | 122 | (Bishop-Lilly *et al.* 2014) |
| ICE*Vch*YB8E08** | *Vibrio cholerae*  YB8E08 | Marine (Oyster pond) | USA/2009 | NA | LBGN01000012 | 104 | (Orata *et al.* 2015) |
| ICE*Vch*YP6E07 | *Vibrio cholerae*  OYP6E07 | Marine (Oyster pond) | USA/2009 | RMS, heavy metal resistance, AAA family ATPase | NMTB01000014 | 93 | Direct submission |
| ICE*Vch*3272-78 | *Vibrio cholerae*  3272-78 | Water | USA/1977 | Ac^r^, Tc^r^, Multidrug transporter, transcriptional regulator, ToxR. | [MIOZ01000052](https://www.ncbi.nlm.nih.gov/nuccore/MIOZ01000052) | 104 | Direct submission |
| ICE*Vch*3223-74 | *Vibrio cholerae*  3223-74 | Storm drain | Guam/1974 | Tc^r^, Ac^r^, transcriptional regulator ToxR, AraC family transcriptional regulator | [MIZG01000083](https://www.ncbi.nlm.nih.gov/nuccore/MIZG01000083) | 104 | Direct submission |
| ICE*Vmy*CAIM528** | *Vibrio mytili*  CAIM528 | Seawater | Spain/1985 | RMA, abortive phage resistance system, XRE family transcriptional regulator | [JXOK01000005](https://www.ncbi.nlm.nih.gov/nuccore/JXOK01000005) | 67 | Direct submission |
| ICE*Vpa*Chn25 | *Vibrio parahaemolyticus*  CHN25 | Marine (Shrimps) | China/2011 | Su^r^, Sm^r^, Tc^r^, Tm^r^, RMS | CP010883 | 88 | (Zhu *et al.* 2017) |
| ICE*Vpa*S163** | *Vibrio parahaemolyticus*  S163 | Marine (Seafood) | Malaysia/2007 | NA | AWHQ01000004 | 76 | (Cui *et al.* 2015) |
| ICE*Vpa*S167** | *Vibrio parahaemolyticus*  S167 | Environment | China/2007 | NA | [AWHM01000024](https://www.ncbi.nlm.nih.gov/nuccore/AWHM01000024) | 82 | (Cui *et al.* 2015) |
| ICE*Vpa*UCM493 | *Vibrio parahaemolyticus*  UCM-V493 | Sediment | Spain/2002 | Beta-lactamase, RMS, transcriptional regulator, XRE, Heat stock proteins | CP007004 | 111 | (Kalburge *et al.* 2014) |
| ICE*Vvu*SC9729** | *Vibrio vulnificus*  SC9729 | Seawater | South Korea/2011 | Cobalt import ATP binding protein | [JZEQ01000086](https://www.ncbi.nlm.nih.gov/nuccore/JZEQ01000086) | 85 | Direct submission |
| ICE*Vvu*CladeA158** | *Vibrio vulnificus*  CladeA-yb158 | Tilapia Fish | Israel/2005 | AraJ, RMS, XRE family transcriptional regulator. | LBNN01000013 | 89 | (Danin-Poleg *et al.* 2015) |
| ICE*Vvu*CG100** | *Vibrio vulnificus*  CG100 | Oyster | Taiwan/1993 | AraJ, LysR family transcriptional regulator, MFS transporter | [PDGD01000031](https://www.ncbi.nlm.nih.gov/nuccore/PDGD01000031) | 105 | (Roig *et al.* 2018) |
| ICE*Vpa*Mal1 | *Vibrio parahaemolyticus*  PCV08-7 | Seafood | Malaysia/2008 | As^r^, RMS | NZ_AOCL01000005 | 71 | (Tiruvayipati *et al.* 2013) |
| ICE*Vpa*Mal2 | *Vibrio parahaemolyticus*  ND24 | Marine | Malaysia/2017 | As^r^, RMS | NZ_POAW01000003 | 79 | Direct submission |
| ICE*Vpa*Mal3 | *Vibrio parahaemolyticus*  ND22 | Marine | Malaysia/2017 | As^r^, RMS | NZ_POAY01000002 | 77 | Direct submission |
| ICE*Vpa*Mal4 | *Vibrio parahaemolyticus*  ND19 | Marine | Malaysia/2017 | As^r^, RMS | NZ_POBB01000003 | 77 | Direct submission |
| ICE*Vpa*Mal5 | *Vibrio parahaemolyticus*  ND16 | Marine | Malaysia/2017 | As^r^, RMS | NZ_POBE01000002 | 77 | Direct submission |
| ICE*Vpa*Mal6 | *Vibrio parahaemolyticus*ND13 | Marine | Malaysia/2017 | As^r^, RMS | NZ_POBH01000002 | 77 | Direct submission |
| ICE*Vpa*Mal7 | *Vibrio parahaemolyticus*  ND12 | Marine | Malaysia/2017 | As^r^, RMS | NZ_POBI01000004 | 77 | Direct submission |
| ICE*Vpa*Mal8 | *Vibrio parahaemolyticus*  ND11 | Marine | Malaysia/2017 | As^r^, RMS | NZ_POBJ01000003 | 77 | Direct submission |
| ICE*Vpa*Mal9 | *Vibrio parahaemolyticus*  NA1 | Marine | Malaysia/2017 | As^r^, RMS | NZ_POBW01000001 | 77 | Direct submission |
| ICE*Vpa*Mal10 | *Vibrio parahaemolyticus*  NA2 | Marine | Malaysia/2017 | As^r^, RMS | NZ_RQNT01000003 | 77 | Direct submission |
| ICE*Vpa*Mal11 | *Vibrio parahaemolyticus*  ST17.P5-S1 | Marine | Malaysia/2017 | As^r^, RMS | NZ_PJOR01000002 | 77 | Direct submission |
| ICE*Vpa*Thd1 | *Vibrio parahaemolyticus*  NCKU_TV_3HP | Marine | Thailand/1999 | As^r^, RMS | NZ_JPKS01000041 | 79 | (Yang *et al.* 2014) |
| ICE*Vpa*Thd2 | *Vibrio parahaemolyticus*  NCKU_TV_5HP | Marine | Thailand/1999 | As^r^, RMS | NZ_JPKT01000008 | 77 | (Yang *et al.* 2014) |
| ICE*Vpa*Vie01 | *Vibrio parahaemolyticus* M1-1 | Marine | Vietnam/2014 | As^r^, RMS | NZ_PDDQ01000009 | 79 | (Kumar *et al.* 2018) |

**Abbreviations associated with this table:**

**AAA**: ATPases Associated with diverse cellular activities **, Ac^r^** : Acriflavin resistance , **Af^r^**: Actiflavine resistance**, Ag^r^**: Aminoglycoside resistance, **Am^r^**: Ampicillin resistance, **Amk^r^** :Amikacin resistance**, Ap^r^**: Apramycin resistance, **As^r^:** Arsenic resistance, **Azm^r^:** Azithromycin resistance, **Az**^r^: Aztreonam resistance, **A/S^r^**: Ampicillin-sulbactam resistance, **Bm^r^**- Bicyclomycin resistance, **Blm^r^**: Bleomycin resistance, **Co**: Cobalt**, Cd**: Cadmium **, Cfz^r^:** Cefazolin resistance **Ctx^r^**: Cefotaxime resistance**, Cip^r^**: Ciprofloxacin resistance**, Cli^r^**: Clindamycin resistance**, Cm^r^**: Chloramphenicol resistance, **Cu^R^**: Copper resistance, **Em^r^**: Erythromycin resistance, **Flo**^r^: Florfenicol resistance, **Fm^r^**: Fosfomycin resistance, **Fq^r^**: Fluoroquinolone resistance, **Hg^r^**: Mercury resistance, **Hm^r^**: Hygromycin resistance, **Km^r^**: Kanamycin resistance, **Lm^r^**: lincomycin resistance, **Ma^r^**: Macrolide resistance, **Nal^r^**: Nalidixic acid resistance, **Nm^r^**: Neomycin resistance, **Nf^r^**: Norfloxacin resistance, **Rif^r^**: Rifampicin resistance, RND: Resistance Nodulation Cell Division, RMS: Restriction modification system, Spt^r^: Spectinomycin resistance, Sm^r^: Streptomycin resistance, **Sum^r^:** Sulphonamide resistance, **Su^r^**: Sulfamethoxazole resistance, **SXT**: Sulfamethoxazole: Trimethoprim resistance, **Til^r^**: Tilmicosin resistance**, Tc^r^**: Tetracycline resistance, **Tm^r^**: Trimethoprim resistance, **To**^r^: Tobramycin resistance, **Zn**: Zinc. **N/A**: Not Available
